# Real particle physics analysis by UK secondary school students using the ATLAS Open Data: an illustration through a collection of original student research

**DOI:** 10.1140/epjp/s13360-024-05518-z

**Published:** 2024-09-02

**Authors:** Eimear Conroy, Alan Barr, Ynyr Harris, Julie Kirk, Emmanuel Olaiya, Richard Phillips

**Affiliations:** 1 Department of Physics, University of Oxford, Parks Road, Oxford, OX1 3PJ UK; 2 Particle Physics Department, Rutherford Appleton Laboratory, Harwell, Didcot, OX11 0QX UK; 3 Institute for Research in Schools, Wellcome Wolfson Building, 165 Queen’s Gate, London, SW7 5HD UK

## Abstract

Since the 2020 release of $10 \hbox { fb}^{-1}$ of integrated luminosity of proton–proton collision data to the public by the ATLAS experiment, significant potential for its use for youth engagement in physics and citizen science has been present. In particular, this article aims to address whether, if provided adequate training and resources, high school students are capable of leveraging the ATLAS Open Data to semi-autonomously develop their own original research projects. To this end, a repository of interactive Python Jupyter notebook training materials was developed, incrementally increasing in difficulty; in the initial instalments no prior knowledge of particle physics or Python coding is assumed, while in the latter stages students emulate the steps of a real Higgs boson search using ATLAS data. This programme was implemented in secondary schools throughout the UK during the 2022/23 academic year and is presented in this article through a collection of research projects developed by a selection of participating students.

## Preamble

During the 2022/23 academic year, a repository of interactive Python Jupyter notebook training materials for meaningfully interacting with the ATLAS Open Data were rolled out to schools across the UK by the UK-based Institute for Research in Schools (IRIS), an organisation which aims to promote original research performed by secondary school students. This is accomplished by connecting and supporting schools and teachers with projects compiled by academic and industry partners, spanning topics from ionic liquid chemistry to Earth observation. This ATLAS Open Data programme and set of resources were developed over the span of three years, including alpha and beta tests with groups of students participating in the International Particle Physics Masterclass at the University of Oxford and with a pilot of approximately 50 students in 6 UK schools in the 2021/22 academic year, respectively.

**Fig. 1:**
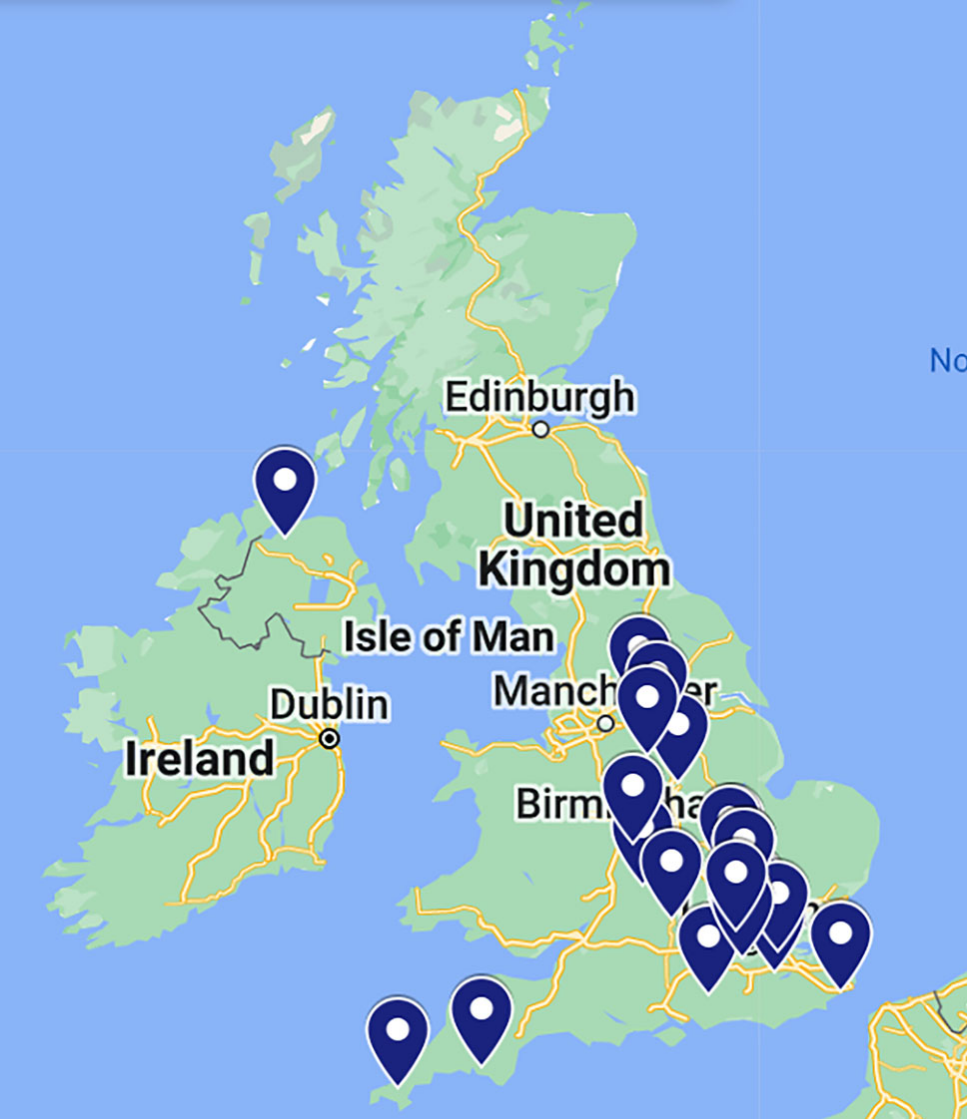
A map illustrating the distribution of all UK schools participating in the IRIS ATLAS Open Data project in the 2022/23 academic year

The full programme was rolled out to approximately 300 participating students from 25 participating schools nationwide in the 2022/23 academic year, where students worked through the repository of notebooks semi-autonomously in small groups, supported by their teachers, who were in turn supported by IRIS and a team of particle physics researchers at the University of Oxford and the Rutherford Appleton Laboratory (RAL). A map illustrating the distribution of participating schools across the United Kingdom is shown in Fig. 1. In general, schools participated for several months to one full academic year. The time invested per week was at the discretion of individual schools and teachers; an example of a common arrangement was a weekly after-school club. With each notebook incrementally increasing in difficulty, the decision was left to the students and their teachers, after which notebook they wished to consolidate their learning and produce an original research project.

The intended research output of these original student projects was a set of posters, with a subset to be presented at one of the IRIS Student Conferences in summer 2023. The target audience of these conferences are the students participating in every IRIS-supported project in a given year, of which the ATLAS Open Data project is one. These conferences allow students to experience an academic conference setting, to present talks and posters to their peers, and ask to questions of their peers’ work in turn. Additionally, following the conference, participating student groups are invited to write proceedings.

This article presents the collection of conference proceedings prepared by participating students in the ATLAS Open Data project, structured such that the order in which the students’ projects are presented approximately the topic order of the repository of notebooks provided to them. All work and writing in Sects. 2–8 is the students’ own, with the exception of framing prefaces written in *italics* and light-touch editing for legibility.

The contributed pieces represent solely the work and perspectives of the secondary school students by whom the individual works were produced, and do not represent the viewpoints of the ATLAS Collaboration.

## Introduction

To date, ATLAS experiment [1] at the Large Hadron Collider [2] has released a total integrated luminosity of 10 $\hbox {fb}^{-1}$ of $\sqrt{s}=13$ TeV proton–proton (*pp*) collision data and corresponding Monte Carlo (MC) simulations [3] to the public, in addition to $1\hbox { fb}^{-1}$ of $\sqrt{s}=8$ TeV *pp* collision data and corresponding MC [4], in accordance with the ATLAS Open Data Access policy [5]. Alongside the release of the data, provided in ROOT format [6], the ATLAS Collaboration has provided extensive documentation for the public datasets and a variety of tools for their usage, targeting secondary school students, undergraduates, graduate students, and the teachers and lecturers who supervise them. One such example is a collection of Jupyter notebooks [7] provided on the ATLAS Open Data portal [8], containing examples of Python data analysis activities and, exploiting the visual advantages of the Jupyter notebook format, accompanying text and images.

**Fig. 2:**
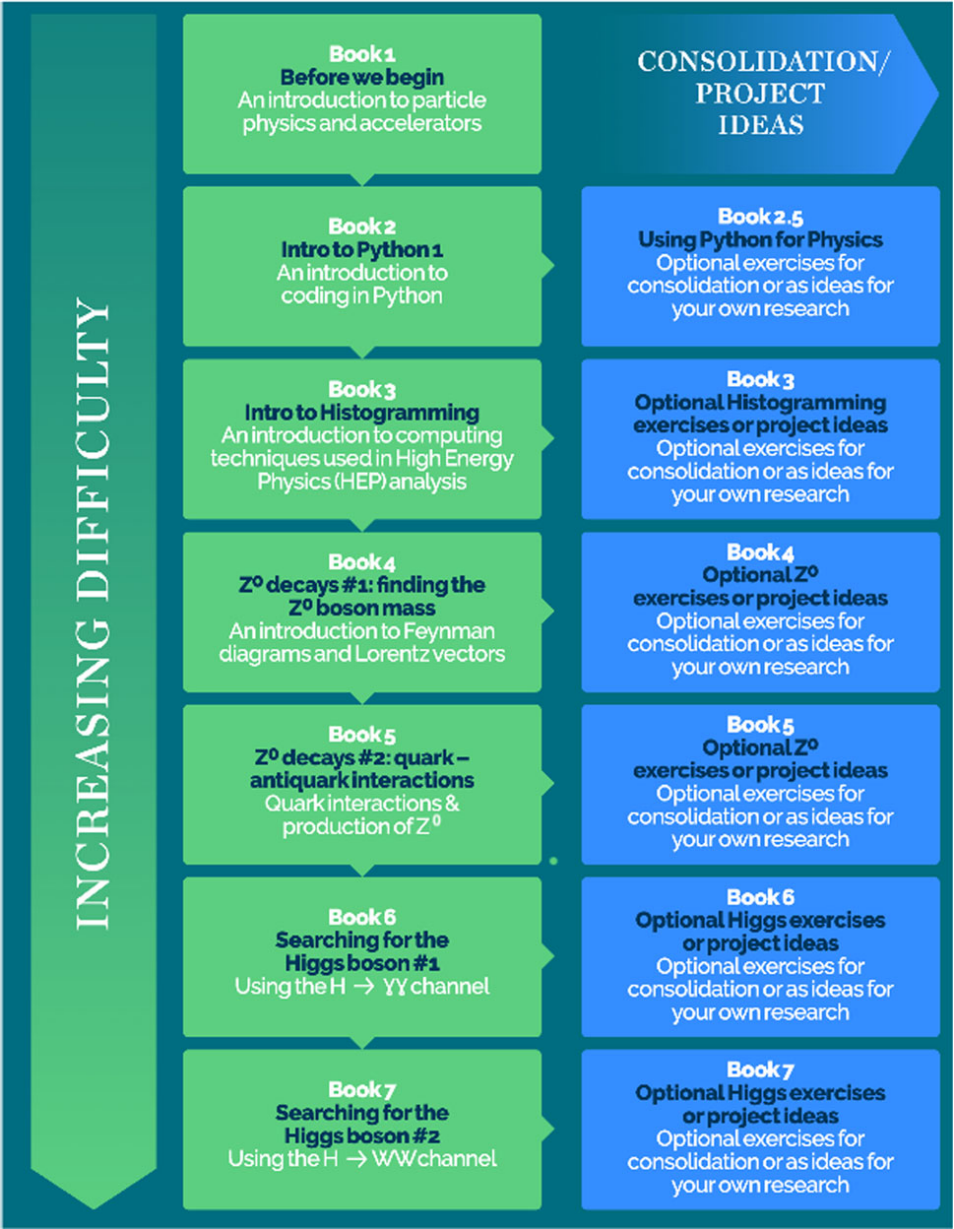
The structure and topics covered by the repository of training materials created for UK secondary school students

In this project, we build on the collection of Jupyter notebooks provided by ATLAS to create a repository [9] of training materials targeting secondary school students in the UK, adding notebooks introducing students to python coding, comprehensive background information for each exercise, extensively commenting example code, integrated exercises with solutions for students, and prompts for learning extension exercises which may also serve as ideas for independent student projects. We aim to address whether, if provided the correct training and resources, secondary school students can produce original particle physics research using the ATLAS Open Data. The created repository comprises a set of seven Jupyter notebooks and supplementary materials, with no prior knowledge of Python coding or particle physics assumed. We take a scaffolded approach; each notebook becomes incrementally more challenging than the previous. The topics covered by each notebook are shown in Fig. 2.

The structure of each individual notebook is as follows:

- Introduction of learning goals;
- Necessary background information presented in a variety of formats—text, diagram, video;
- Worked data analysis example, including accompanying text and substantively commented code;
- “Try it yourself” exercise. Particularly in early notebooks, the code structure is provided, with blanks left to be filled by students;

At the conclusion of each notebook, prompts for learning consolidation exercise/ independent project ideas are provided. These “off ramps” are chosen such that they may be completed using only the skills acquired to this point, providing flexibility to students and teachers with respect to the time and resources they wish to invest. Sections 2–8 present reports of these independent projects in the students’ own words, with the order selected to roughly follow the right-hand (blue) column of Fig. 1 so that this article reflects the structure of the repository of training materials.

## Developing python coding skills to model projectile motion and analyse data from high-energy collisions in the Large Hadron Collider

By students of **Limavady Grammar School**: Leo Collins, Darcy Cooper, Ella Feeney, Callum Gilpin, Emily Harnett, Annabelle Hunter, Norman Ling, Rebecca McCausland, Aoife McLaughlin, Olivia McLernon, Ryan Wilson.

### Preface

*In this section, the students consolidate skills developed in the first four notebooks of the repository. They rely on background information on the Large Hadron Collider and the ATLAS experiment developed from notebook 1, Python coding skills developed in notebook 2, and the ability to interact with ROOT files to produce histograms of event kinematics developed in notebook 3. They employ these skills to develop a Python model of project motion and compare the benefits of their model with analytical methods learned in school, extract and plot lepton multiplicity in a sample of simulated events, and to reproduce the* *Z* *boson mass peak.*

### Summary

In this project, we developed skills in coding with Python. Using these skills, we were able to write the code necessary to solve projectile motion questions. This provided us with an insight into the advantages and disadvantages of using this method over completing these calculations manually. We then used our Python skills to create histograms via coding. The histograms produced show that, in the chosen samples of simulated data with no selections applied, it is most likely that only one lepton will be reconstructed in each event inside the Large Hadron Collider. Finally, we applied appropriate selections to the data to reconstruct the *Z* boson invariant mass peak. Via the use of Python code, we determined the mass of the *Z* boson to be approximately equal to 90 GeV.

### Aims

1. To use Python programming language to write a programme to find the distance travelled by a 10 kg projectile fired at 15 m/s at $45^\circ$ above the horizontal from a point 2 m above the ground;
2. To use Python programming language to display data in the form of a histogram of simulated high-energy collisions in the Large Hadron Collider;
3. To use Python to analyse data from high-energy collisions in the Large Hadron Collider and determine the mass of the *Z* boson.

### Background information

#### Python coding

Python is a popular general-purpose programming language that can be used for a wide variety of applications. It is used to build websites, software, and perform analysis [10]. Python also offers the ability to easily automate processes through scripting, making it key for software testing, troubleshooting, and bug tracking. It plays a key role in data science tasks and is used to perform complex statistical calculations, visualise data, and create machine learning algorithms [11].

Python is used today in research to solve many of the world’s complex modern physics problems. However, it may also be applied to more basic physics equations, for example, calculations involving constant velocity. This can be done by simulating the motion of an object. If we know the object’s *x* positional coordinate at a particular time *t* and its instantaneous velocity *v* along the *x*-axis at that time, this will allow us to find the object’s *x* position a small time later $\Delta t$ by substituting these values equation:

1 $$\begin{aligned} x_t+\Delta t = x_t+v\Delta t. \end{aligned}$$

Similarly, Python can be used to solve basic problems of constant acceleration, by updating Eq. 1 to reflect that, since the object is now accelerating, its velocity is changed at every step and must be updated accordingly:

$$\begin{aligned} x_t+\Delta t = v_t+a\Delta t. \end{aligned}$$

#### Projectile motion

Projectile motion is the motion of an object thrown or projected into the air, experiencing only the acceleration due to the force of gravity. The object is called a projectile, and its path is called its trajectory [12]. The resultant path is in effect a combination of two motions—horizontal and vertical. This allows us to apply the equations of motion separately in each orthogonal direction. Particles in a projectile follow a curved path known as a parabola.

#### The Large Hadron Collider

The Large Hadron Collider (LHC) [2] is the world’s largest and most powerful particle accelerator. It consists of a 27-kilometre ring of superconducting magnets with a number of accelerating ‘radio frequency cavity’ structures to boost the energy of the particles along the way [13].

#### ATLAS

The ATLAS detector [1] is a general-purpose particle physics experiment at the LHC at CERN. ATLAS is the largest detector of its kind and is designed to record the high-energy particle collisions of the LHC, which take place at a rate of 40 million interactions per second in the centre of the detector. The ATLAS Collaboration is a large global collaboration with the common aim of better understanding the fundamental constituents of matter and their interactions [14]. The ATLAS project investigates a wide variety of fundamental particles, from the Higgs boson to what makes up dark matter [15].

#### Fundamental particles

There are two types of fundamental particles, quarks and leptons. Each of these groups have six particles, related in pairs. The six quarks are paired into three generations; the up and down quark, the charm and the strange quark; and the top and bottom quark. The six leptons are also arranged into three generations; electrons and electron neutrinos, muons and muon neutrinos, and the tau and tau neutrino [16]. The electron, muon and tau lepton all have an electric charge and a sizeable mass, whereas the neutrinos are electrically neutral and have very small mass. There are four fundamental forces, three of which result from the exchange of particles called bosons. The *W* and *Z* bosons are responsible for the weak force [16].

The *Z* boson is very unstable and does not live long enough to be detected, so to find the *Z* boson we reconstruct it from its decay products. We will reconstruct *Z* bosons which have decayed into two leptons. To conserve charge and lepton number these leptons need to have an opposite charge and the same flavour, meaning we will be looking for a muon and an antimuon or an electron and an antielectron (positron).

### Aim 1

#### Method

To code the simulation for a projectile motion, we used the following method:

- Set the initial conditions of the projectile;
- Make a loop over time steps;
- In the loop, update the velocity of the projectile (only its y-coordinate changes).

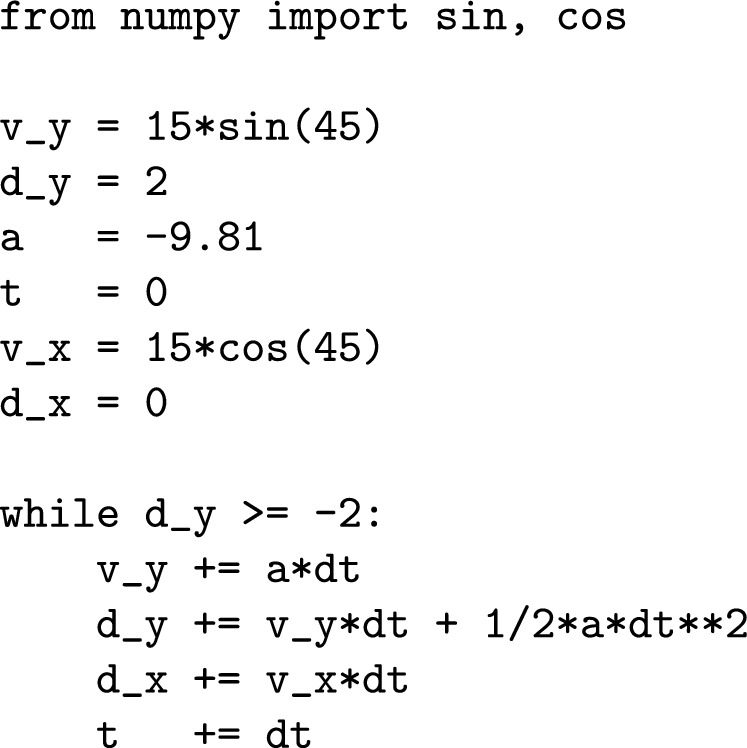

#### Results

**Fig. 3:**
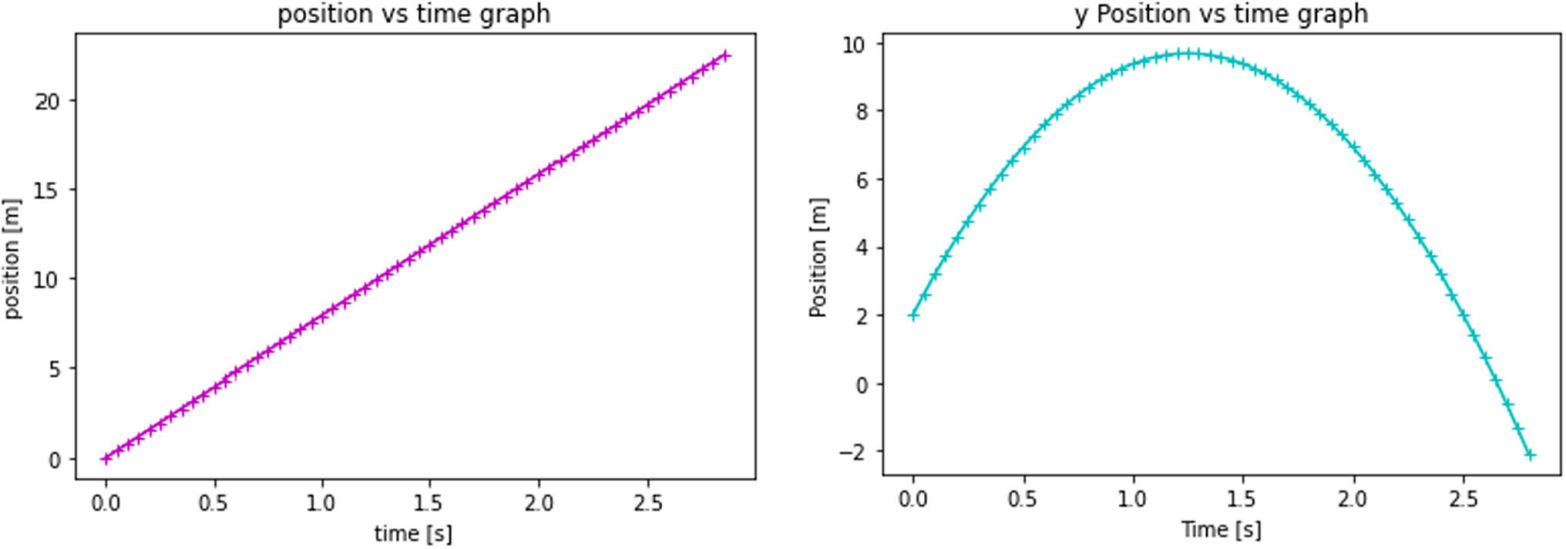
Graph showing the projectile *x* position against time (left) and *y* position against time (right)

From Fig. 3, it can be determined that the ball hits the ground, −2 m below its starting position, at time t= 2.80 s. At this time, it is at a horizontal distance of 22.1 m from its starting point, moving with a vertical velocity of − 14.7 m/s and a horizontal velocity of 7.9 m/s.

#### Analysis and conclusion

Advantages of simulating projectile motion using Python:

- Good method of graphically displaying projectile motion;
- Efficient technique for analysing large amounts of data;
- Once the code is correct there is no room for human error;
- Provides a clear representation of calculations.

Disadvantages of simulating projectile motion using Python:

- Requires a comprehensive understanding of Python code;
- May be difficult to apply to more complex physics problems.

### Aim 2

#### Method

1. Load a ROOT file containing MC simulation from the ATLAS Open Data [17] database by using the Python uproot library;
2. To fill histogram, extract the number of leptons from our TTree using uproot. A TTree is a container that keeps track of the information from a collision event. Fill the histogram using the .fill() function from the Python hist module;
3. Plot the histogram using the .plot() and plt.show() functions from the Python matplotlib library;
4. Title the histogram and create a labelled *x*-axis.
5. Normalise the histogram so that it shows the proportion of each number of leptons produced, up to a maximum value of 1, instead of the absolute number of collision events that produced the different numbers of leptons.

#### Results

**Fig. 4:**
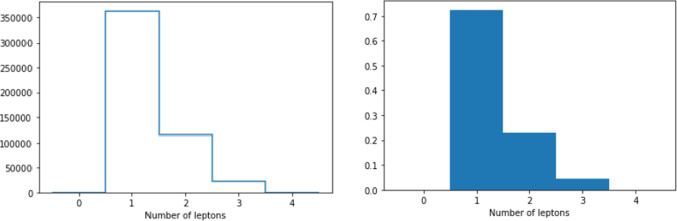
A histogram showing the absolute number of leptons produced per collision event (left) and the same histogram normalised to 1 (right)

Figure4 shows the lepton multiplicity of simulated events accessed as described above. The strong peak around at 1 shows that the majority of open data events included have one lepton, a result which was expected given that the histogram was produced from a sample with a one-lepton inclusive filter applied.

#### Analysis and conclusion

An advantage of displaying large datasets in histogram format is that the information can be viewed in a clear, concise way, allowing for the major features of the distribution of the data to be seen. From the MC simulation analysed, we were able to see trends in many leptons were produced in each collision event simulated in that sample. The histograms produced show that, most commonly, only 1 lepton is produced per collision event.

### Aim 3

#### Method

1. First, open a ROOT file of data collisions file using uproot, and inspect the contents of the file;
2. Use the .arrays method of uproot to import only specific variables for each event, and then define a histogram, with *x*-axis named mass/GeV;
3. Make cuts in the data [17], requiring two leptons of the same flavour, and then cut the data again requiring that those two leptons are oppositely charged, to reconstruct the *Z* boson invariant mass;
4. Import the Python matplotlib module and plot the histogram.

#### Results

**Fig. 5:**
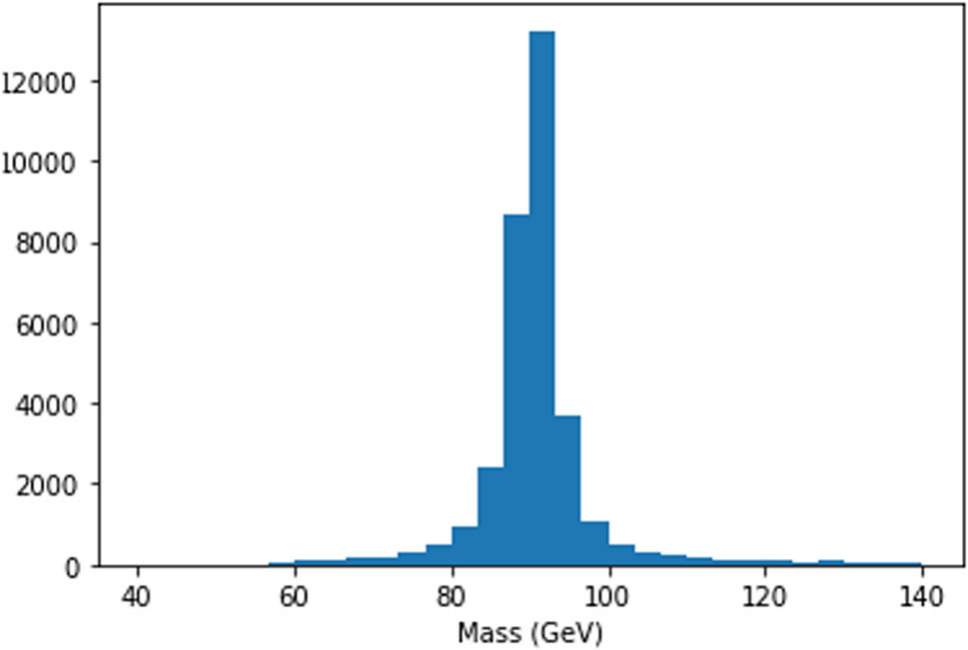
Graph showing the mass distribution of a *Z* boson

Figure 5 shows the mass of the particles fitting the above criteria. The strong peak at around 90GeV shows that this is the mass of the *Z* boson.

#### Analysis and conclusion

The $qq\rightarrow Z\rightarrow ll$ process is not the only way in which *Z* can be produced at the LHC; it is possible for virtual interactions between quarks and antiquarks to produce two *Z* bosons which then both decay in the same way as mentioned above to create a final state with four leptons. Exploring this interaction could be an alternative method to determine the mass of the *Z* boson.

## An investigation in to energy conservation in the decay of the *Z* boson

By students of **Lady Manners School**: Caleb Byrne, George Colver.

### Preface

*In this section, students consolidate skills developed in notebooks 4 and 5, reproducing* *Z* *boson mass peaks in events with two or four leptons in the final state through the application of object and event selections and reconstructing the four-momentum of pairs of leptons. The students also explore the impact of statistical concepts, such as sample size and bin width, on particle physics results.*

### Summary

In this article, we discuss our processes for reconstructing the mass of a *Z* boson by analysing the four-momentum of the decay products. This has been achieved by analysing events from the ATLAS Open Data [17, 18], recorded (and simulated) by the ATLAS experiment [1] CERN. A number of filtering conditions were used to identify cases where *Z* bosons had decayed into lepton–antilepton pairs, and these events were analysed to determine the invariant mass of the original unstable particles. These evaluations were done using the Python programming language, and the results were presented as histograms that display the frequency density for a range of masses; the modal peak can be taken as the true mass of the *Z* boson.

### Introduction

**Fig. 6:**
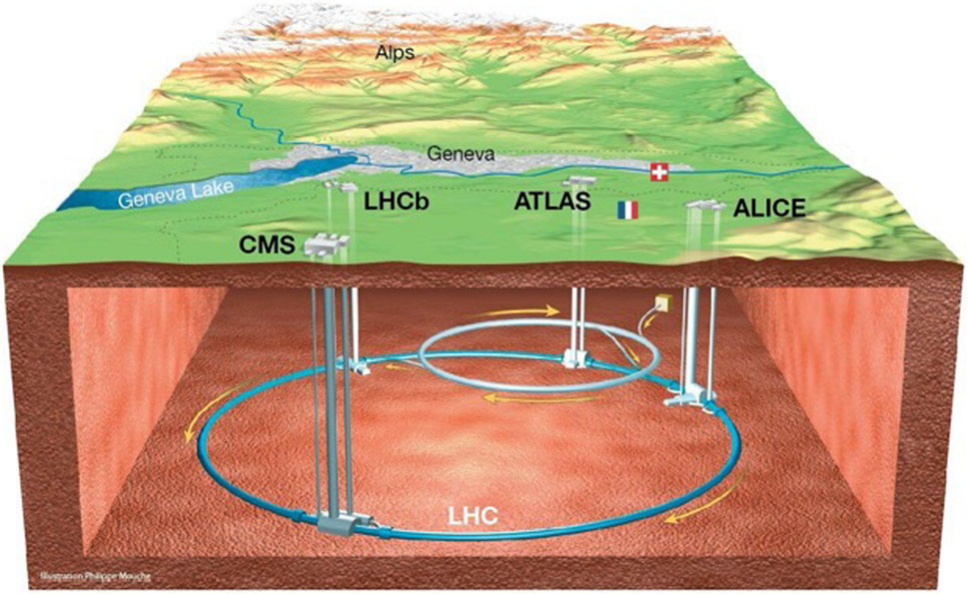
The Large Hadron Collider, a depiction of the four detectors present at the LHC: ATLAS, LHCb, ALICE and CMS [19]

The ATLAS Experiment [1] is one of four experiments located at the Large Hadron Collider [2] (shown in Fig. 6), where protons are accelerated to relativistic speeds and collided together, producing a great number of particles, including the *Z* boson.

The ATLAS Experiment consists of four main subsystems, each concerned with identifying and measuring the properties of different particles as they pass through. Moving outward from the collision point, these are:

- The silicon Tracker shows the paths taken by charged particles, allowing the momentum of these particles to be calculated by analysing the curvature of their trajectories;
- The Electromagnetic Calorimeter measures the energy of particles which interact electromagnetically, such as photons and electrons, by recording the electric signals produced by their passage through layers of liquid argon and dense absorber material;
- The Hadronic Calorimeter measures the energy of strongly interacting hadrons using layers of steel in which the particles are stopped;
- Finally the Muon Spectrometer measures the energy and momentum of muons, which pass through the previous layers interacting very little.

**Fig. 7:**
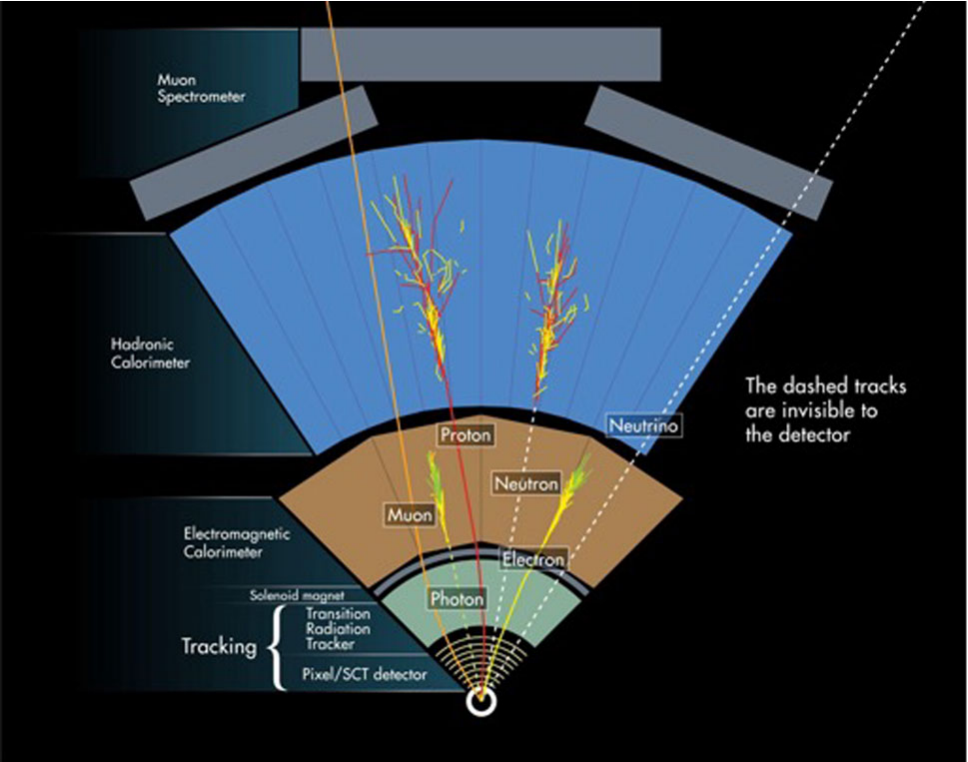
A slice of the different sub-detectors within the ATLAS detector [20]

The layers described above are shown in Fig. 7.

As highlighted in Fig. 7, particles cannot be detected until they reach the relevant layers of the detector. This can be a problem if a particle is unstable and prone to decay, like the *Z* boson, as it is not possible to detect directly. Instead, the particles that it decays into must be considered, and used to reconstruct the original particle.

**Fig. 8:**
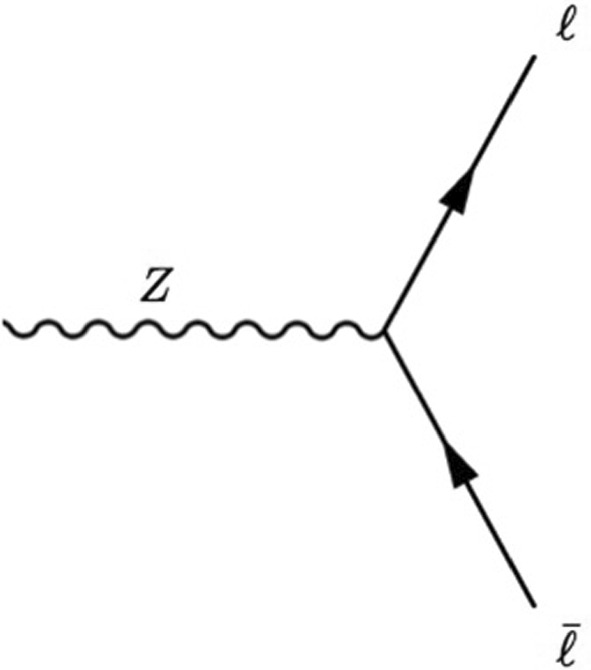
A Feynman diagram of *Z* boson decaying into a lepton–antilepton pair

The *Z* boson can decay in a few different ways [21]. The most common ($\sim$70% of decays) channel is the *Z* decay into hadrons; however, as these particles can be produced by a number of processes within the accelerator, this is not the cleanest possible signature to accurately reconstruct the *Z* boson. The second most common decay route ($\sim$20% of decays) is the decay into neutrinos. However, since neutrinos are incredibly weakly interacting, they can only be indirectly inferred from Missing Energy in an event. Finally the last decay route ($\sim$10% of decays) produces a lepton–antilepton pair, show in Fig. 8, and this channel suits our criteria of being easily measurable, and a unique enough event to identify accurately.

**Fig. 9:**
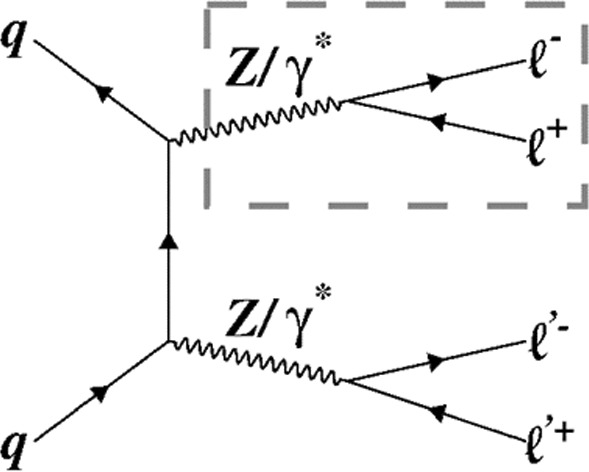
Quark interaction, producing two unstable *Z* bosons

By also considering another channel, we can be yet more stringent in our selection of events. It is possible for two quarks to interact, exchanging a virtual particle, and both decaying into *Z* bosons which then decay via the channels already identified, as shown in Fig. 9. This means that two different lepton–antilepton pairs are produced, each within the window of the *Z* boson mass, a more unlikely coincidence if they were produced by another process, allowing for a more confident identification of these events as being the decays of *Z* bosons.

Once the criteria for an event containing a *Z* boson decay had been determined, we were able to use these events to reconstruct the mass of the *Z* boson.

### Method

Due to the laws of conservation of mass-energy and momentum, the total momentum and mass-energy of the lepton–antilepton pair produced must be equal to the total momentum and mass-energy of the *Z* Boson that produced them. We used a Lorentz vector to store these mass-energy/momentum values concisely, sum them, and finally calculated the invariant mass of the original *Z* Boson. The structure of a Lorentz vector is shown below:

$$\begin{aligned} P = (E,p_x,p_y,p_z), \end{aligned}$$

The *E* component is the mass-energy of a particle, and the *p* components are the momentum in each of the three spatial directions. These vectors can be combined by simply adding the relevant elements, and once the resultant vector is known, the invariant mass can be calculated as shown below:

$$\begin{aligned} m = \frac{1}{c^2}\sqrt{E^2-p^2c^2} \end{aligned}$$

Using these tools, the invariant mass of a *Z* boson can now be calculated. We used a mixture of real, recorded events from the ATLAS Open Data [17, 18], and simulated [22] events in our investigation to evaluate the accuracy of the simulations and ensure that our method works in both cases.

This was achieved by first loading the data (using the Python uproot library to load the files into ROOT TTree data structures from which the events could then be extracted), and then iterating through each event, selecting events where the number of leptons present was an even number greater than 0, where the number of positive (antileptons) and negative (leptons) leptons was identical and of the same flavour, and hence where a pair of leptons originating from a *Z* boson was produced. These pairs then had their Lorentz vectors filled and summed to allow the invariant mass of the original *Z* boson to be calculated. These values were then used to fill a histogram, ready for analysis and inspection.

### Results

In order to improve the presentation of our results, we used 60 bins in our histograms, from 60 to 120 GeV. These results are discussed below.

**Fig. 10:**
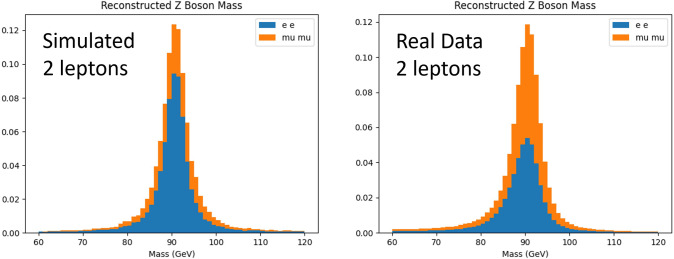
Determining the mass of the *Z* boson in the two-lepton channel using both simulated data (left) and real data (right)

Initially, we determined the mass of the *Z* boson in the two-lepton channel, as shown in Fig. 10. The modal mass peaks on both the left-hand plot, corresponding to simulated *Z* boson decays, and on the right-hand plot, corresponding to real data, suggest a mass for the *Z* boson of 91 GeV $\pm 1$ GeV. Compared to the accepted result of 91.2 GeV, our measurement is in good agreement. The right-hand histogram containing real data events is very similar to the simulated data, suggesting a high degree of accuracy in the CERN simulations. The real data showed considerably more $Z\rightarrow \mu \mu$ decays; however this is likely due to aspects of the particular data files we chose to analyse such as trigger thresholds or object filters, and not an actual discrepancy.

**Fig. 11:**
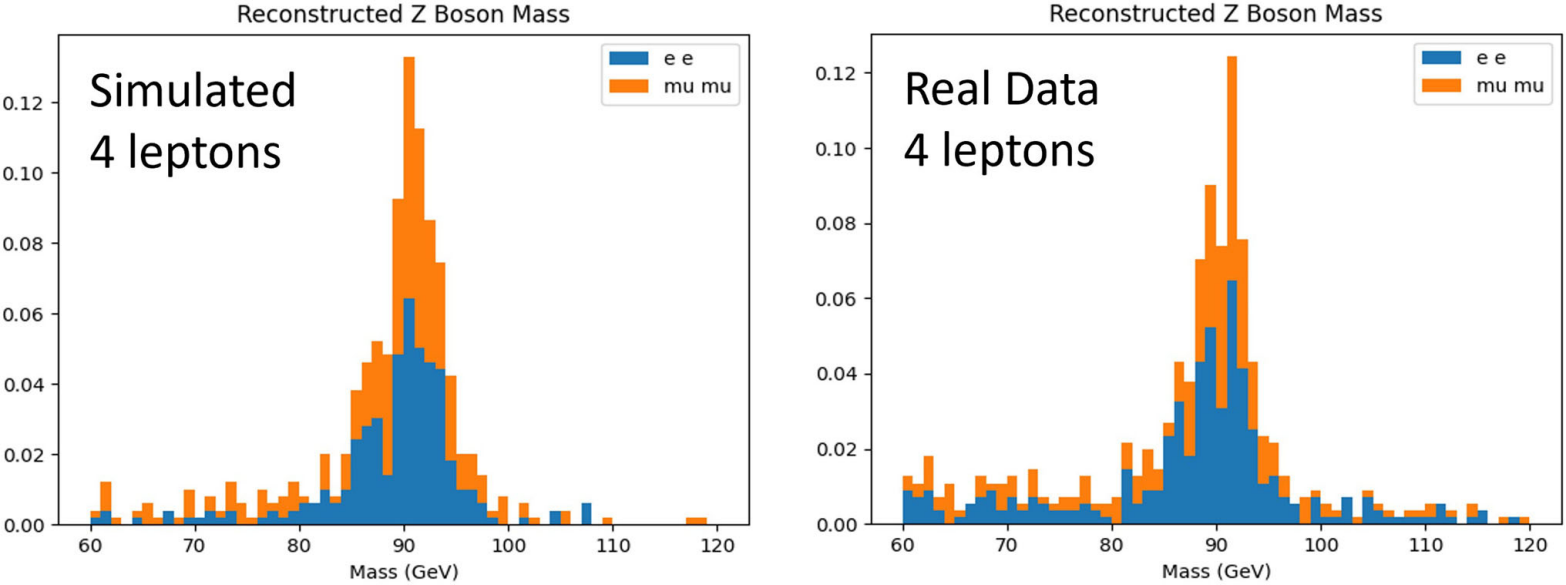
Determining the mass of the *Z* boson in the four-lepton channel using both simulated data (left) and real data (right)

Subsequently, we repeated our measurement of the *Z* boson mass in the four-lepton channel, as shown in Fig. 11. For both the simulated (left) and real data (right) invariant mass distributions for the decays of two *Z* bosons to a total of four leptons are rather similar to those in the two lepton channel shown in Fig. 10; however, it is considerably less ‘clean’, tending to show more spikes at lower masses and unusual dips at $\sim$88 GeV.

### Analysis and conclusions

All histograms we generated from a large sample of data sets indicate a *Z* boson mass of $\sim$90 GeV, which is very close to the accepted value of 91.2 GeV [21], giving credence to our methodology.

**Fig. 12:**
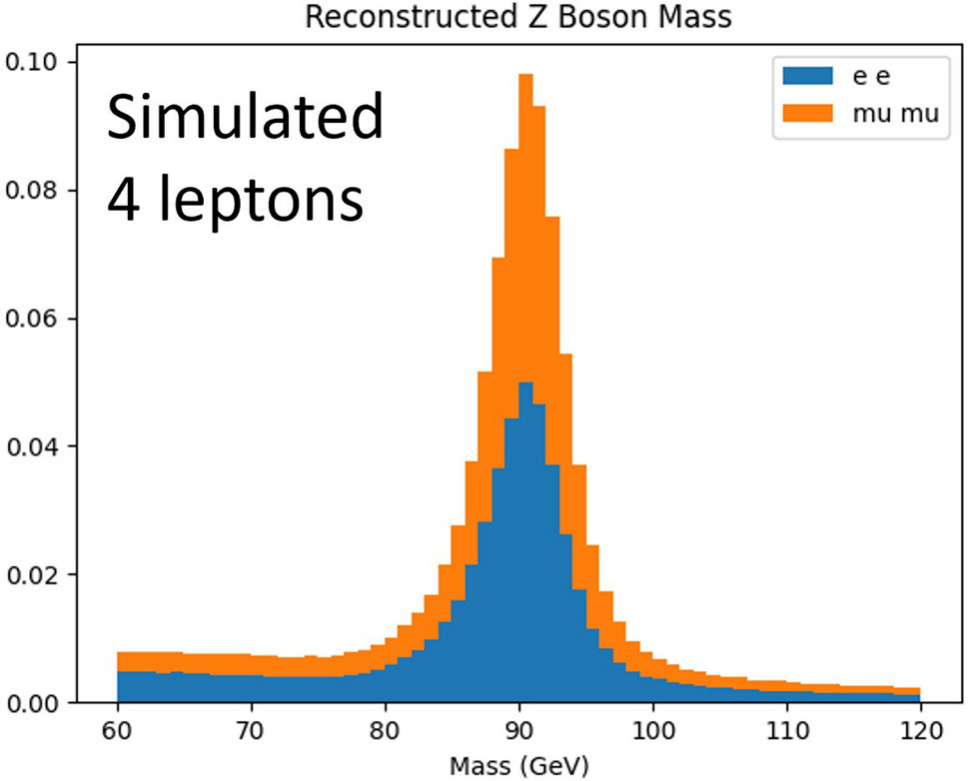
Use of a large data set for decays producing 4 leptons produced a smoother histogram

Upon reflection, we realised that the reason the distributions created by the events involving four leptons were less well-fitted to a bell curve was because the data sets containing these events that we were initially using were orders of magnitude smaller than the equivalent two lepton datasets; random errors and fluctuations were magnified, and the distribution formed by these events was subsequently far less smooth. Using a larger data set for decays producing four leptons produces a far smoother histogram, as shown in Fig. 12.

### Further investigation

Our research so far has provided us with some interesting and exciting results, and we plan to continue to investigate the concept of detecting particles by working backwards from their more stable decay products to determine information about the original, less stable particle. We plan to apply similar methods to those discussed here to study other particles such as high mass quarks, and other bosons such as the *W* and Higgs bosons. Looking at the theory behind more complex interactions and planning experimental procedures to glean information about them is also an exciting concept which we plan to explore in the future.

### Acknowledgements

The research and findings presented in this article could not have occurred without the help of IRIS, the University of Oxford, RAL, Dr Neil Garrido (Regional Schools Engagement Lead), and Dr Ebbens (Lady Manners Head of Physics).

## Investigation into experimental methods

By students of **Lady Manners School**: Eleanor Joinson, Robbie Milton.

### Preface

*In this section, students consolidate skills developed in notebooks 6 and 7, to search for the Higgs boson using two different methods; a bump hunt in the* $H\rightarrow \gamma \gamma$ *channel, and a non-resonant search in the* $H\rightarrow WW$ *channel. In the former, the students explore the concept that larger datasets lead to clearer signal peaks, while in the latter, ideas such as irreducible backgrounds and the use of simulation are explored.*

### Introduction

Peter Higgs and Francois Englert won the Nobel Prize for Physics in 2013 for their work on the Higgs boson. In 1964, Higgs submitted a paper which predicted the existence of the Higgs field, which allowed boson mass to be introduced to the Standard Model. To test for the Higgs field, the Large Hadron Collider (LHC) [2] was used to search for a Higgs ’particle’ associated with the Higgs field, which would be unstable. In 2012, the evidence gathered by the LHC at the ATLAS detector was sufficient and strong enough to officially ’discover’ the Higgs boson [23, 24].

We decided to investigate how different statistical methods for searching for new particles are more convincing than others, and how the accuracy and validity of identical data can alter based upon the tests applied. The two tests we conducted were via the $H\rightarrow \gamma \gamma$ channel, where the Higgs boson will decay into two photons resulting in a larger concentration of photons of the known mass of the Higgs boson, and the $H\rightarrow WW$ channel which is tested using a non-resonant search, where the small amount of difference in the transverse masses [25] between the data and predicted backgrounds provide evidence for the Higgs boson. This allowed us to compare the two methods in terms of statistical significance so that the strength of the different tests could be evaluated.

### Method 1

**Fig. 13:**
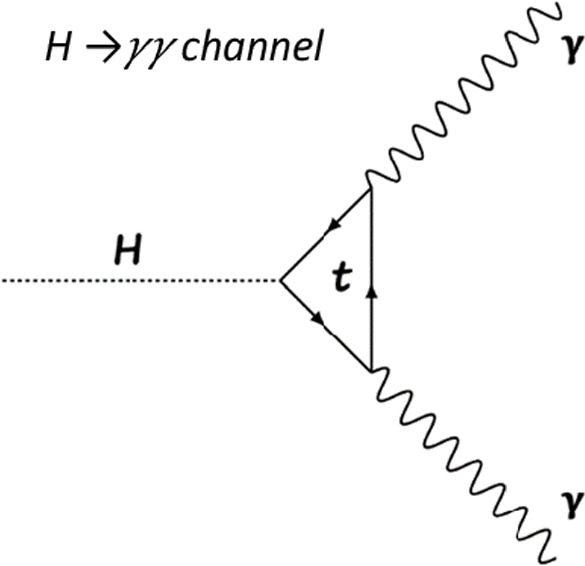
$$H\rightarrow \gamma \gamma$$ channel

The method used to first prove the existence of the Higgs boson involved the $H\rightarrow \gamma \gamma$ channel shown in Fig. 13; however, diphoton pairs very commonly produced in LHC collisions, which means that only after analysing billions of collisions can a clear ’bump’ in the otherwise continuous curve of the diphoton invariant mass produced. It is also impossible to know the exact collisions the Higgs boson was produced in, but the significant increase in the number of diphoton events around the mass of the Higgs boson is enough evidence to confidently prove its involvement.

Using the four diphoton data sets available in the ATLAS Open Data [26], each containing millions of events, we selected the relevant photon objects that met ’Tight’ requirements. Our requirements for a Tight photon were: Event passes photon trigger, photon object is reconstructed, photon has $p_{\mathrm{T}}>$ 25 GeV, photon is in the ’central’ region of ATLAS ($|\eta |<$ 2.37), photon does not fall in the ’transition region’ of ATLAS (1.37 $\le |\eta |\le$ 1.52) between the calorimeter barrel an endcap.

**Fig. 14:**
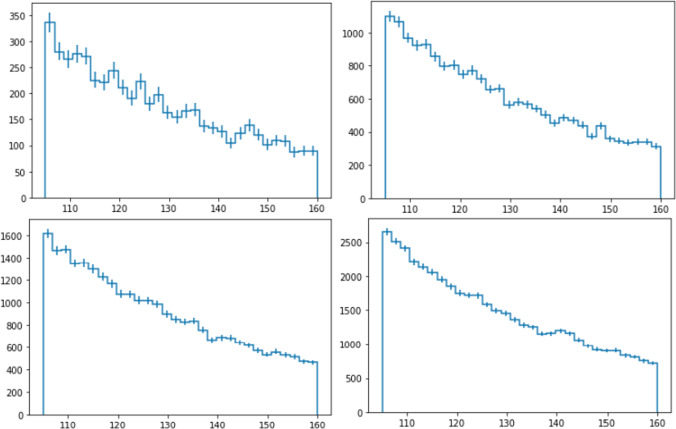
Histograms showing diphoton invariant mass distributions for each of the four available data sets

Once the good-quality photons were extracted, Lorentz vectors of their four-momenta were built, and their respective invariant masses were calculated. From each data set, we produced a histogram showing the diphoton invariant mass, shown in Fig. 14.

**Fig. 15:**
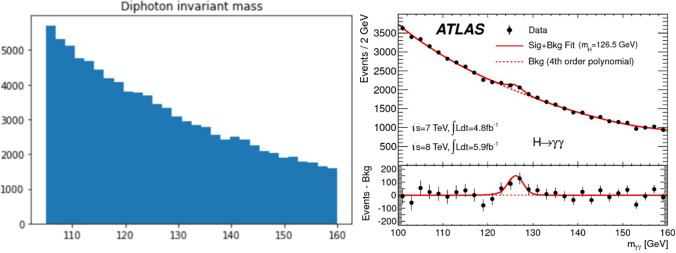
Histogram showing diphoton invariant mass for the combined four data sets (left), and a full ATLAS measurement of the $H\rightarrow \gamma \gamma$ channel [27] included for illustrative purposes (right)

These histograms were then combined to produce a single histogram showing the data from all sets in one graph, shown in the left-hand side of Fig. 15. While not directly comparable due to differences in event weighting, an example plot from a full $H\rightarrow \gamma \gamma$ analysis is shown for illustrative purposes on the right-hand side of Fig. 15.

The full data show a clear ‘bump’ in the data at 125 GeV when compared to the fourth-order polynomial, which shows the predicted trend without the Higgs boson. We see a similar ’bump’ in our histogram, but it is not as clear as the ATLAS results. However, we do still produce data with a deviation from the predicted data trend at the known mass of the Higgs boson. This suggests that while our data are less conclusive than the full ATLAS data, it still shows some evidence of the Higgs boson.

### Method 2

**Fig. 16:**
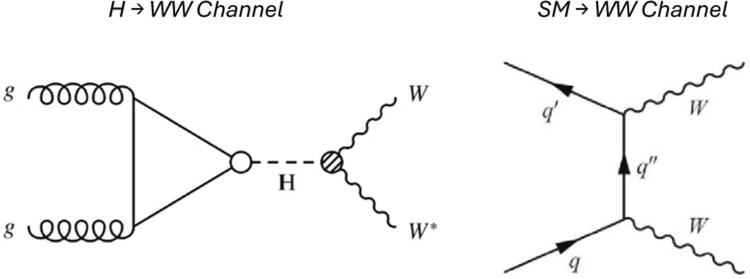
Feynman diagram of the Higgs boson decaying to 2 *W* bosons signal (left), and the Standard Model diboson background production from two quarks (right)

In addition to the $H\rightarrow \gamma \gamma$ channel, the $H\rightarrow WW$ channel, shown in Fig. 16 is an alternative method used to prove the existence of the Higgs boson. This method is tested using a non-resonant search, where we investigate the difference between the background prediction (created using simulations) and the data [28], with any remaining events after the background is subtracted indicating the presence of the Higgs boson. This search therefore relies heavily on accurate simulations.

**Fig. 17:**
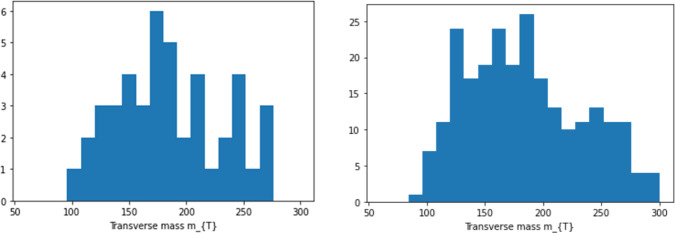
Histogram of the raw transverse mass frequencies of one data set without the removal of the background diboson production (left), compared to the remaining events after the simulated background data is removed (right). The resulting ‘left-over’ events are evidence for the presence of the Higgs boson

The simulated background must be scaled to ensure the sample size is in proportion to the number of data events recorded. Then, ’good leptons’ must be selected to improve the signal-to-background ratio of the events we selected. These leptons must pass ’Tight’ requirements: The lepton must be isolated and central ($|\eta |<$2.37). If the event shows exactly two leptons (indicative of a $H\rightarrow WW$ decay, with each lepton originating from a leptonic *W* boson decay) then their Lorentz vectors are created and so the transverse masses [25] can be calculated (using the .Mt() function which came inbuilt with the training Jupyter notebooks) and plotted on a histogram, showing the frequency of different transverse mass values, shown in Fig. 17 (left).

We repeated this process for the predicted background data. From there, we subtracted the simulated background from the data, shown in Fig. 17 (right). This showed a clear excess of events, that can then be explained by the existence of the Higgs boson.

### Analysis and conclusions

Both the experiments that we conducted resulted in viable evidence for the existence of the Higgs boson. In the first experimental method, the histogram produced does show a slight deviation from the 4th-order polynomial curve seen over the rest of the data in a similar place (at 125 GeV) to the full ATLAS data. The reduced clarity of the ’bump’ can be explained by the much smaller data set available for us to use, as the production of the Higgs boson is a rare event.

The second method we conducted uses simulated data to help identify the Higgs boson, by removing the ’expected’ events present, assuming no Higgs boson, from the ’actual’ events recorded to observe some events that were previously unaccounted for. Due to the use of simulations and scaled predicted results, the data might not give as conclusive evidence for the presence of the Higgs boson, as the results were not generated directly from raw data. This is in contrast to the first experimental method, in which all data points were filtered and selected from the raw data collected by ATLAS.

As a result, we concluded that the first method, the $H\rightarrow \gamma \gamma$ channel, yielded more convincing results showing good agreement with the known mass of the Higgs boson, even with the comparatively smaller data set.

Moving on with our research, we plan to investigate other channels of Higgs boson decay, continuing to evaluate the evidence produced. From there, we would look to refine the tests further, with the aim of improving the validity of the results, and how convincing the evidence for the existence of the Higgs boson would be.

## Searching for the Higgs Boson through the $\gamma \gamma$ and $W^+W^-$ decay channels

By students of **City of London Academy Highgate Hill**: Dan Trinder, Elani Ponnampalam, and Radhe Das.

### Preface

*In this section, students exploit techniques developed in notebook 4* ($Z\rightarrow ll$ *search*), *notebook 5* ($ZZ\rightarrow 4\,l$ *search*), *notebook 6* ($H\rightarrow \gamma \gamma$ *search*) *and notebook 7* ($H\rightarrow WW$ *search*) *to explore their original ideas in analysis code development, validation and optimisation. The* *Z* *boson mass peak is used to validate analysis code before searches for the Higgs boson are conducted. Additionally, the students develop original data caching and multithreading mechanisms to increase the efficiency of their data processing.*

### Summary

The primary focus of this study was to detect the Higgs boson through exploration of the $H\rightarrow \gamma \gamma$ and $H\rightarrow WW$ decay channels. Utilising the publicly released 13 TeV proton–proton collision data recorded by the ATLAS experiment [1], we employed Python modules and libraries such as NumPy, pickle and uproot to analyse over 10 million events. In addition to this, we developed two new modules: the rootFile module, which was created to combine data from various sources, and a multithreading module, which accelerated data processing. We refined our analysis code initially by testing it on a simulated sample Standard Model $Z\rightarrow l^+l^-$ and $ZZ\rightarrow l^+l^-l^+l^-$ decays in the ATLAS detector, provided by the ATLAS Open Data [18, 28]. Our analysis of the Standard Model simulation relied on two foundational principles in physics: the conservation of invariant mass and the conservation of momentum. Following this, the $H\rightarrow \gamma \gamma$ decay channel [26], was explored using a technique known as ‘bump hunting’, and for the and $H\rightarrow WW$ decay channel [28], we conducted a non-resonant search. Despite rigorous analysis of the ATLAS Open Data to which we had access, our results were inconclusive; we did not detect the Higgs boson.

### Introduction

In 2012, the ATLAS [1] and CMS [29] collaborations detected the Higgs boson at the Large Hadron Collider (LHC) [2], decades after it was first theorised in 1964 [30]. This discovery not only confirmed the existence of the Higgs field—which is responsible for a particle’s mass—but also marked the beginning of a continuing effort to understand its properties [31]. These endeavours may shed more light on phenomena like dark matter, which makes up most of the universe’s mass content but remains undetected [32]. This study aims to contribute to this understanding through exploration of two of the most common decay channels of the Higgs boson: $H\rightarrow \gamma \gamma$ and $H\rightarrow W^+W^-$. We employ an iterative approach; refining our code as the study progresses, as well as sophisticated computational techniques such as multithreading to accelerate data analysis. With this research we aim to play a role in furthering scientific understanding of the Higgs boson.

### Method

Given the unstable nature of the Higgs boson, we focused on studying its decay products rather than the particle itself, due to its short lifetime. By utilising two fundamental laws in physics: the conservation of invariant mass [33] and the conservation of four-momentum, it was possible to reconstruct the Higgs boson from selected decay channels, and study it further.

Using the publicly released collection of 13 TeV proton–proton collision data recorded by the ATLAS Experiment, we began stage one of our validation process. The first step was to filter out events that contained more than two leptons, then to further reduce the data by eliminating pairs that did not have the same flavour and opposite charge. We then combined their individual four-momenta and used this to calculate their reconstructed invariant mass. At this stage of our research, we began the development of a more sophisticated file loading system—a prototype of our later rootFile module—and incorporated a data caching mechanism to reduce the impact of any computational errors that may have occurred and caused us to lose significant amounts of progress.

Following this, we modified our lepton event data filtration criteria to discard events that did not have four leptons. Similarly to the previous stage, we removed lepton pairs that did not have two leptons with opposite charge and same flavour. In addition to this, we implemented a for loop that matched and analysed every possible lepton pair, then selected the two pairs that, based on their invariant mass, were most likely to have originated from a *Z* boson decay and plotted them on the histogram.

After these two validation steps, we explored the $H\rightarrow \gamma \gamma$ decay channel, employing Python code and ROOT Lorentz vectors to reconstruct the invariant masses of the decay products of the Higgs boson, and examining the resultant histogram to determine whether we had succeeded in detecting the Higgs boson. A ’bump’ around 125 GeV—the invariant mass of the Higgs boson—would have confirmed its presence. Considering that we were detecting photons in this stage, not leptons, we updated the filtering criteria. To be counted as a valid event, the photon had to pass ‘tight’ requirements, be well isolated, pass the photon trigger, and have sufficient transverse momentum. The invariant masses of photon pairs that that satisfied each of those requirements were plotted on a histogram along with error bars displaying the statistical uncertainty in our measurement. In addition to this, we did a data-driven estimate of the background diphoton events and fitted the resulting cubic function to our histogram to make the $H\rightarrow \gamma \gamma$ events easier to see. We also created a multithreading module to speed up the data processing, after realising that the analysis of 10 million events would take a substantial amount of time and utilised the revised rootFile module to make the data extraction more efficient.

During our exploration of the $H\rightarrow WW$ decay channel, we conducted a non-resonant search. This is done by plotting the transverse mass [25] of the $W^+W^-$ for experimental data recorded by the ATLAS experiment, then subtracting the transverse mass of Monte Carlo simulations of background events coming from the SM *WW* diboson background production from two quarks and plotting the result on a histogram. *W* bosons cannot be detected directly in the ATLAS detector because they are unstable and decay too quickly. Instead, we look at their decay products—a lepton and missing energy from a neutrino—to confirm the *W* bosons’ presence. In addition to this, each simulated event had to be scaled to account for the greater number of events in the Monte Carlo sample than in the ATLAS data. After subtracting the simulated backgrounds, we plotted the resultant histogram, which displayed our measured Higgs signal.

### Results

The focus of our study was the exploration of two common decay channels of the Higgs boson: $H\rightarrow \gamma \gamma$ and $H\rightarrow W^+W^-$. Prior to investigating these decays, we carried out two stages of preliminary tests to ensure our code was reliable.

#### Code validation using $Z\rightarrow \mu ^-\mu ^+$ and $Z\rightarrow e^- e^+$

**Fig. 18:**
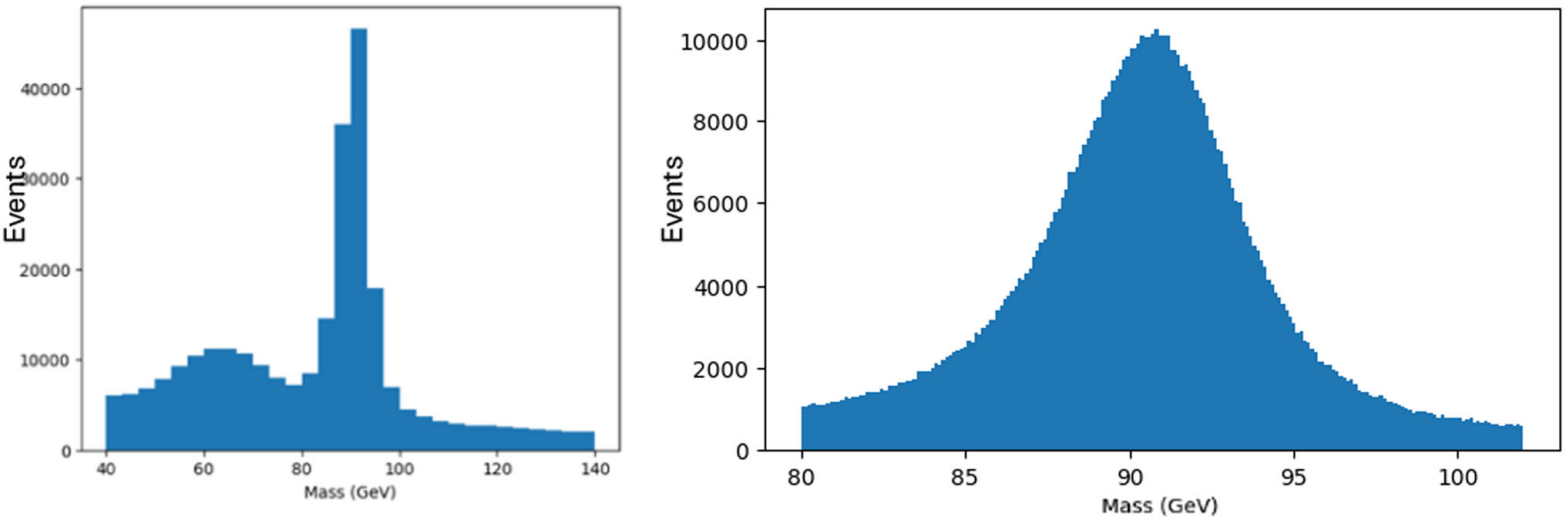
Code validation through observing an event cluster at the invariant mass of the *Z* boson (91±5 GeV). The reconstructed invariant masses of both $e^+e^-$ and $\mu ^+\mu ^-$ pairs were plotted, both before (left) and after (right) an additional cut on dilepton invariant mass, to highlight the *Z* peak

To validate the accuracy of our code, we began by testing by detecting *Z* bosons using dilepton event data recorded using the ATLAS experiment at CERN from the ATLAS Open Data collection [28]. To confirm our code was functional, we would need to see a significant cluster of events around 91 GeV—the invariant mass of the *Z* boson [34]. Figure 18 (left) shows the results after initial cuts were made based on lepton flavour and charge, and, as expected, the resulting histogram had a high concentration of events with a reconstructed mass within the range of $86-96$ GeV ($91\pm 5$ GeV). To gain a clearer view of the mass distribution, we removed lepton pairs that fell outside this mass range and increased the bin count, resulting in Fig. 18 (right). This observation of the *Z* peak in pairs of leptons with a combined invariant mass of approximately 91 GeV confirmed the reliability of the code.

#### Code validation using $qq\rightarrow ZZ\rightarrow l^+l^-l^+l^-$

**Fig. 19:**
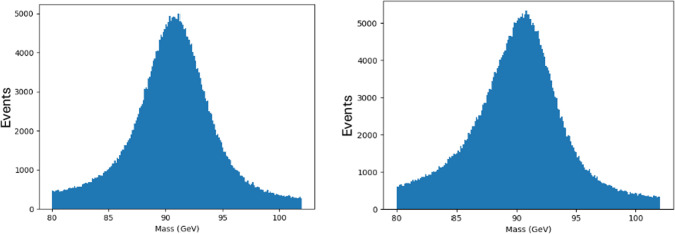
*ZZ* production in the four-lepton channel. The invariant masses of $e^+e^-$ and $\mu ^+\mu ^-$ pairs are plotted separately for $e^+e^-$ (left) and $\mu ^+\mu ^-$ (right) pairs

To further validate the accuracy of our code, we instead analysed four-lepton events [18]. As before, to confirm that our code was reliable, we needed to see that a large fraction of the lepton pairs had an invariant mass within the range $86-96$ GeV ($91 \pm 5$ GeV). We were expecting to plot a histogram with a similar shape to that of Fig. 18. Figure 19 displays the reconstructed masses of same-flavour, opposite-sign lepton pairs in events with four leptons in the final state, separately for $e^+e^-$ (left) and $\mu ^+\mu ^-$ (right). Each plot has the *Z* peak shape we were expecting to see. Thus, we were able to conclude that our code was functional and that we had a solid foundation for further analysis.

#### Searching for the Higgs Boson: $H\rightarrow \gamma \gamma$

**Fig. 20:**
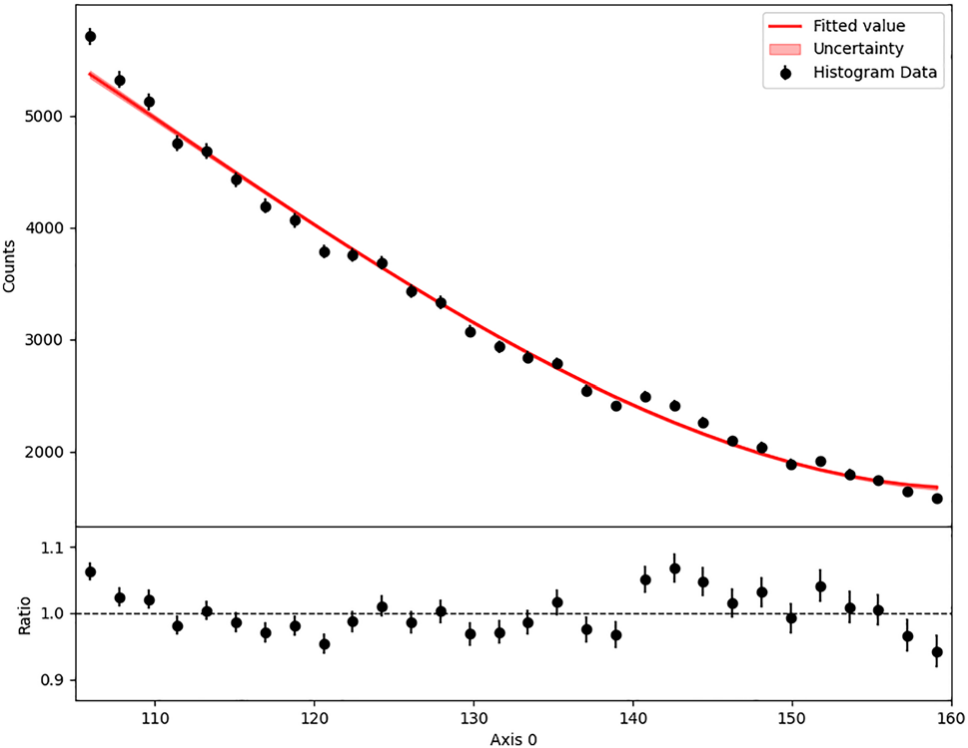
A technique called ‘bump hunting’ was employed to detect the Higgs boson when conducting analysis of the $H\rightarrow \gamma \gamma$ decay channel. We fitted a cubic function representing a data-driven estimate of background diphoton events (the red line) and normalised it (the grey line) to attempt to make the bump easier to see

Having validated the reliability of the code, we explored the decay channel $H\rightarrow \gamma \gamma$ [26]. Figure 20 depicts our results. Unfortunately, our analysis did not show a significant ’bump’ around the expected value of 125 GeV (the invariant mass of the Higgs boson [35]); therefore, we were unsuccessful in confirming its presence through the analysis of this decay channel.

#### Searching for the Higgs Boson: $H\rightarrow W^+W^-$

**Fig. 21:**
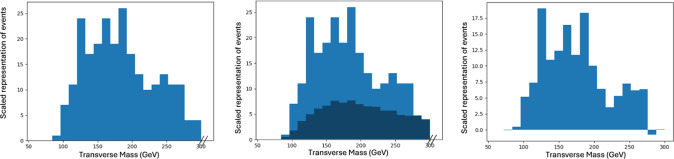
Each stage of performing a non-resonant search for $H\rightarrow WW$ production. Selections are applied to dilepton data (left), $qq\rightarrow WW$ backgrounds are estimated using MC simulation (middle), which were finally subtracted from data to produce our measurement of the Higgs signal (right)

The last stage of our research involved conducting a non-resonant search to detect pairs of *W* bosons from dilepton data [28], shown in Fig. 21 (left). After using Monte Carlo simulations to estimate the background, displayed in Fig. 21 (middle), we subtracted this result from the data, to produce Fig. 21 (right). The presence of a Higgs signal in our resulting histogram suggested the presence of the Higgs boson. However, other factors must be considered before we can conclude that we detected the Higgs boson, e.g. we only accounted for one background process.

### Discussion

#### Code validation using $Z\rightarrow \mu ^-\mu ^+$ and $Z\rightarrow e^- e^+$

Our findings in Stage 1 demonstrate that our code was functional, since it accurately identified and reconstructed *Z* boson events from lepton pairs. As we hypothesised, there was a large concentration of events around 91 GeV, which indicated that the filters we used were effective in isolating events that originated from the decay of a *Z* boson. To make the mass distribution easier to see, we increased the number of bins and introduced a mass cut to filter events that were unlikely to have come from a *Z* boson decay. The results we achieved align well with research conducted by other groups in the past.

After conducting an initial round of analysis, we decided to implement a data caching mechanism in preparation for future stages. The selected events from the ROOT files we had downloaded and merged using the rootFile module were converted into an array and locally cached in a JSON file, exported using the pickle module. This means that if the code needed to be re-run, the JSON file could be loaded instead of going through the process of re-downloading and reanalysing the data, which would be time consuming.

Our results were promising, however, further refinements could be made to both the rootFile module prototype and our analysis code to make the data processing more efficient.

#### Code validation using $qq\rightarrow ZZ\rightarrow l^+l^-l^+l^-$

Stage two extended the validation process; however, rather than applying our code to events with two leptons, we applied it to events with four. This provided us with the opportunity to apply our code to a more complex scenario and check whether it was reliable enough to be used in further stages.

Recognising that by exploring events involving four leptons, we introduced the possibility that a single event could yield up to 4! valid pairs, we implemented a for loop that generated and analysed each of the 24 potential lepton pairs. Any pair that did not satisfy all the filters was discarded, and following this, only the pair that had a reconstructed mass closest to that of the *Z* boson (91 GeV), was included in the histogram. Although incorporating this step into our methodology extended the time taken for the data analysis, it proved worthwhile as it enabled us to identify a greater number of valid lepton pairs. Similarly to our approach during the initial stage, we employed the rootFile module to increase the efficiency of our analysis chain. We iteratively refined this module as we progressed to ensure it was fully functional by the time we began searching for the Higgs boson.

The results we achieved showed that our code was robust. There was a large peak of events around 91 GeV ($\pm 5$ GeV), which is what we hypothesised we would see. Figure 19 closely resembles Fig. 18, which we had previously established was accurate. This strongly suggests that our code was reliable and could be used as the foundation for our data analysis in stages 3 and 4.

#### Searching for the Higgs Boson: $H\rightarrow \gamma \gamma$

Upon validating our code, the focus of our research shifted to the exploration of the $H\rightarrow \gamma \gamma$ decay channel. As depicted in Fig. 20, the data analysis did not yield the desired result: a ’bump’ around 125 GeV. Despite fitting a cubic function that represented a data-driven estimate of the background diphoton events to our histogram to make the $H\rightarrow \gamma \gamma$ events easier to see, we were unsuccessful in confirming the presence of the Higgs boson.

Several factors could explain this. First, it is possible that our ‘tight’ requirements resulted in the discarding of many ‘good photons’. If we erred too far on the side of caution, trying to ensure that we did not involve events that did not originate from the $H\rightarrow \gamma \gamma$ decay channel in our histogram, we may have missed valid photons originating from a Higgs decay. Not including these photons in our histogram may have resulted in the absence of the ’bump’ at 125 GeV. Secondly, we may not have analysed enough events to see the concentration of events around the invariant mass of the Higgs boson. $H\rightarrow \gamma \gamma$ is a rare event and is often difficult to see over any background diphoton events. Additionally, the data-driven background fit to a cubic function may not have been robust enough to make the result visible on the histogram. It is possible that the use of a larger sample size may have resulted in the presence of the ‘bump’ at 125 GeV.

After realising that we had millions of events to analyse, which would take a significant amount of time, we decided to create a multithreading module. This module was made to speed up data processing by utilising threads to divide the workload into smaller sections that could run simultaneously. Instead of analysing each event one by one, multiple events were analysed at once, leading to a significant decrease in the time taken for the data processing.

Although we were unsuccessful in detecting the Higgs boson, we made improvements to the efficiency of our code through the development of the multithreading module, which proved especially beneficial for Stage 4 of our research.

#### Searching for the Higgs Boson: $H\rightarrow W^+W^-$

To search for the Higgs boson through analysis of the $H\rightarrow W^+W^-$ decay channel, we conducted a non-resonant search. This was necessitated by the fact that the masses of the decay products were greater than the invariant mass of the Higgs boson, thus rendering the ‘bump hunting’ technique we had previously used not applicable. By subtracting the Monte Carlo simulated background from the real data, we estimated the Higgs signal, as depicted in Fig. 21. Although this method did yield a Higgs signal, and therefore in theory confirmed the presence of the Higgs boson, it should be noted that our histogram only considered one source of background—diboson events. Had we considered multiple sources of background, the resultant histogram would have a much smaller Higgs signal, or possibly not one at all. Therefore, to confirm the presence of the Higgs boson, we would need to conduct further analysis on several other sources of background events, as well as the one we already considered, and re-examine the histogram.

### Conclusions

Our research began with thorough code validation using $Z\rightarrow \mu ^-\mu ^+$ and $Z\rightarrow e^- e^+$ and $qq\rightarrow ZZ\rightarrow l^+l^-l^+l^-$ events, which confirmed the reliability of our code in preparation for subsequent stages. While we did not yield results through exploring the $H\rightarrow \gamma \gamma$ decay channel, we made significant improvements to our code, and developed a multithreading module to expedite data processing. After analysis of the $H\rightarrow W^+W^-$ decay channel, we identified a Higgs signal. However, we could not confirm the presence of the Higgs, as we did not consider enough sources of background events to conclusively say that we detected it. Our iterative approach allowed for incremental improvements, and refining first drafts of code as we progressed through the stages streamlined the analysis process later. Our work demonstrated the benefits of such an approach and highlighted several areas for improvement, for example, the need for a larger sample size when exploring decay channels. Therefore, although our results did not confirm the presence of the Higgs boson, the code we utilised to explore our chosen decay channels provides us with a solid framework for future research endeavours.

## Re-proving the existence of the Higgs Boson

By students of **The Tiffin Girls’ School**: Ayda Yazdani, Soniya Walke, Phoebe Lister.

### Preface

*In this section, students leverage the* $H\rightarrow \gamma \gamma$ and $H\rightarrow WW$ s*earch techniques developed in notebooks 6 and 7 to design a search in an additional decay channel:* $H\rightarrow ZZ$. *This is supported by skills in analysing* *ZZ* *production developed in notebook 5. Students also explore the idea of background fits, implementing an original fit to the continuum background to* $H\rightarrow \gamma \gamma$ *to emphasises the Higgs mass peak.*

### Summary

The Higgs field plays a critical role in the Standard Model of particle physics. All massive elementary particles interact with the field through the Higgs mechanism and acquire mass, enabling them to form matter and give rise to the complex structures one can observe in the universe. The Higgs boson is the mediator the Higgs field.

The experimental method used to discover the Higgs boson involved the precise measurement of the properties of particles produced in proton–proton collisions at the Large Hadron Collider [2] using the ATLAS detector [1], and the use of statistical methods to identify the Higgs boson signal from the background events. We intended to apply these same skills on a smaller scale through our analysis of publicly available ATLAS Open Data [18, 26, 28] datasets from the ATLAS detector available on CERN’s website [8].

In our research, we investigated three decay channels of the Higgs boson: Higgs to two photons, Higgs to two *W* bosons and Higgs to two *Z* bosons. Executing cuts on the collection of ATLAS Open Data allowed us to target each of these channels individually and plot histograms to piece together evidence of the Higgs boson’s existence.

### Introduction

The Standard Model of particle physics describes how the fundamental forces interact with particles. These elementary particles are classified into fermions, or ‘matter particles’, and bosons, known as ‘force carriers’, the latter which includes the Higgs boson.

Half a century after the Higgs mechanism was first proposed in the 1960s by the theoreticians Robert Brout, François Englert, and Peter Higgs, the existence of the Higgs boson was finally confirmed in 2012. Data was collected from the ATLAS [1] and CMS [29] detectors, at the Large Hadron Collider [2] at CERN to prove the existence of the particle. The measured data displayed a deviation from the expected backgrounds: in early ATLAS measurements, the invariant mass distribution of two photons produced in the diphoton channel showed a slight bump near 126 GeV (consistent with the Higgs boson’s hypothesised mass) with a significance of 2.2 standard deviations above the Standard Model (SM) background [36]. Later combinations with CMS results produced an observation of the Higgs boson at 125 GeV over 5$\sigma$, above the threshold for discovery.

### Research aims

**Fig. 22:**
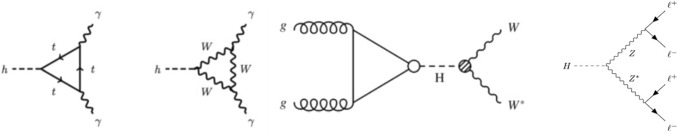
Feynman diagrams for two $H\rightarrow \gamma \gamma$ decay modes (left) [37], one $H\rightarrow WW$ decay mode (middle) [38], and one $H\rightarrow ZZ$ decay mode (right)

This project aimed to re-prove the existence of the Higgs boson by exploring Higgs boson production in three of its most sensitive decay channels, each shown in Fig. 22:

1. The $H\rightarrow \gamma \gamma$ decay channel in the two-photon final state (2.8$\sigma$ local significance observed), looking for evidence of the Higgs boson in distributions of diphoton invariant mass, expecting a mass of 125 GeV;
2. The $H\rightarrow WW$ decay channel in the two-lepton final state (1.4$\sigma$ local significance observed), using a non-resonant search technique;
3. The $H\rightarrow ZZ$ decay channel in the four-lepton final state (2.1$\sigma$ local significance observed), by reconstructing the diboson invariant mass.

### Methods

We used the 13 TeV ATLAS Open Datasets [18, 26, 28], which provided us with data collected from real proton–proton collisions detected by ATLAS, in addition to simulated samples. These data files, presented in ROOT format [6], were analysed using a selection of Python libraries suited to our research: the uproot module for reading in data, the numpy library to carry out statistical analysis and the hist module from the larger matplotlib library for data visualisation and plotting histograms produced from our analyses.

### Results

#### Higgs to diphoton ($H\rightarrow \gamma \gamma$) channel

In order to prove the existence of the Higgs boson through the Higgs to diphoton channel, we needed to plot the invariant mass of the two photons produced in selected event data, chosen after making suitable cuts to ensure that mainly events involving a two-photon system were included. The process, including the cuts involved, is enumerated below.

**Fig. 23:**
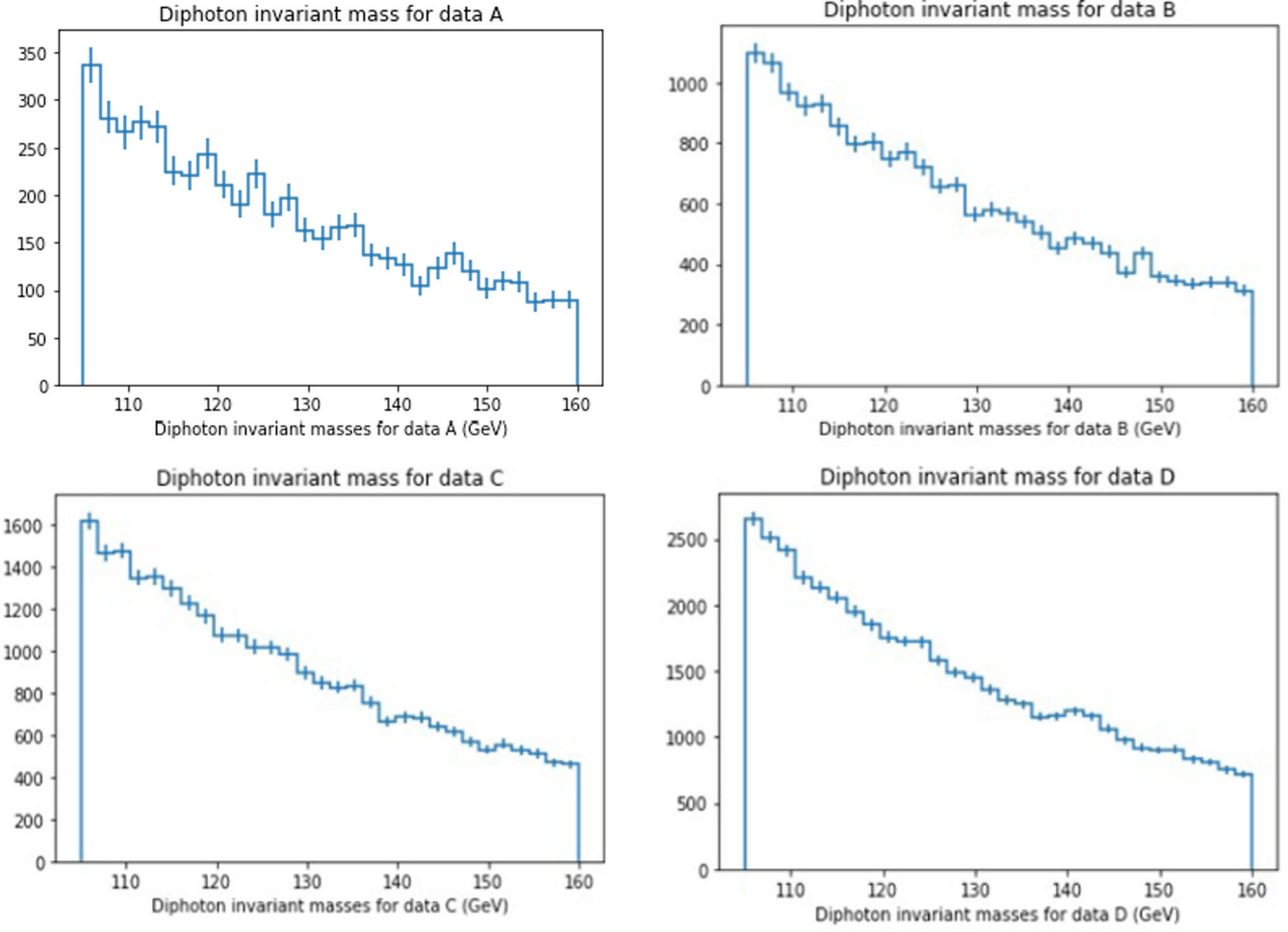
Diphoton invariant mass plots for datasets A-D

1. Loop through each data event in the TTree (a ROOT data storage format) from the ROOT file containing the collision data;
2. In each event, search for good-quality photons, which must:
  Pass the diphoton trigger;
  Pass “Tight” reconstruction requirements;
  Have $$p_{\mathrm{T}}>25$$ GeV;
  Be in the ‘central’ region of ATLAS with $|\eta | < 2.37$, excluding the ‘transition region’ between ATLAS’s Inner Detector barrel and electromagnetic calorimeter endcap $1.37 \le |\eta | \le 1.52$;
3. If the two photons are well-isolated, extract their four-momentum from the $p_{\mathrm{T}}$, $\eta$, $\phi$ and energy, and store them in a TLorentzVector, a ROOT object emulated in the Jupyter notebook resources, which stores the energy and momentum of a particle as a four-vector. A function allowing us to extract invariant mass from a TLorentzVector was also provided;
4. Add the TLorentzVectors associated with the two photons together;
5. Calculate the invariant mass of the two-photon system;
6. Check each photon makes up a minimum fraction of the diphoton system invariant mass;
7. Fill a histogram with the invariant mass of the two-photon system.

We repeated this process for all the diphoton datasets, labelled A–D, provided by the ATLAS Open Data. We plotted separate histograms for each dataset, as shown in Fig. 23.

**Fig. 24:**
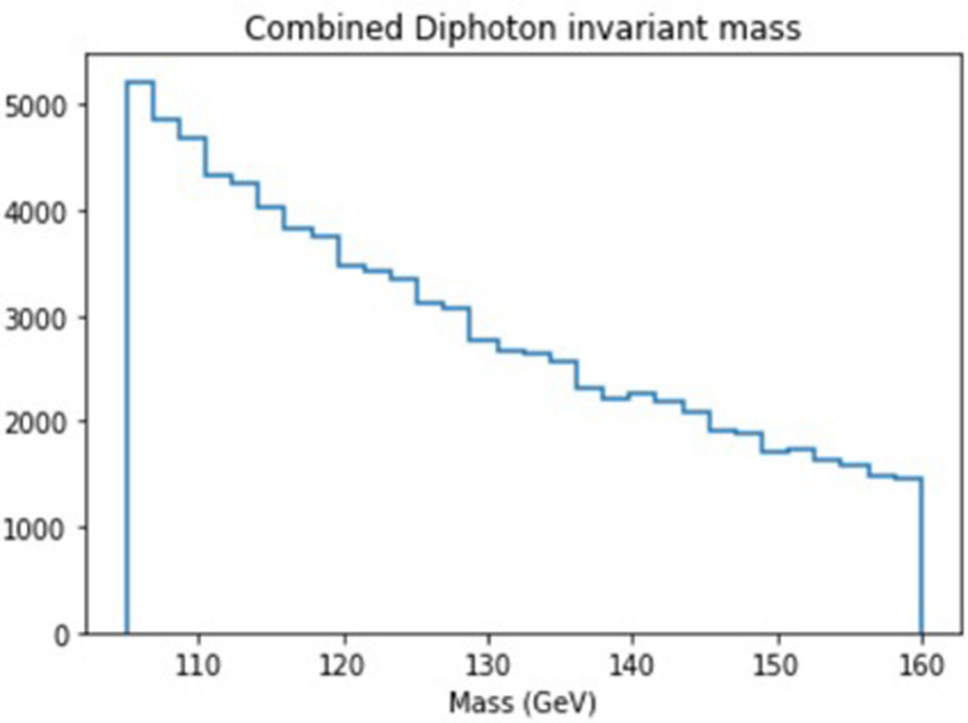
Merged histogram for the diphoton invariant masses obtained from all events in A, B, C and 2,900,000 events of D (ran out of memory)

The datasets had varying numbers of events, with dataset D having the highest number (3,600,000 events), making the processing time longer for this dataset. Due to this, when merging the histograms for the different datasets to make the bump at the Higgs boson mass more visible, we were unable to include all events from dataset D, as we were limited by the processing power of our computers. The merged diphoton invariant mass histogram is shown in Fig. 24.

**Fig. 25:**
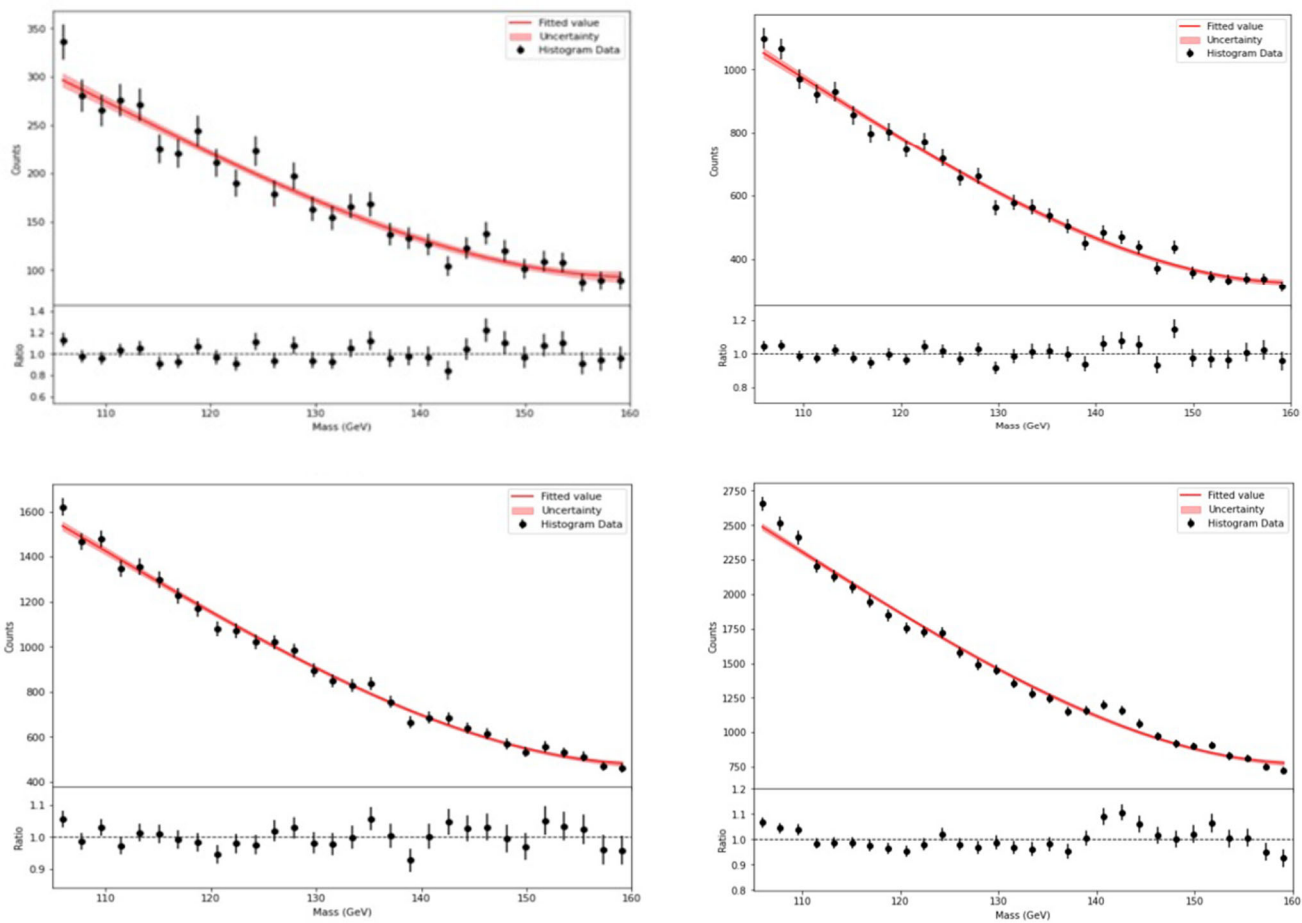
Fitted histogram for datasets A-D

To make the Higgs bump clearer to see, we produced a prediction of the background using a cubic function to fit the graph and plotted the data against this to make it stand out more. The background fits to each individual dataset are shown in Fig. 25.

#### Higgs to two *W* bosons ($H\rightarrow WW$) channel

**Fig. 26:**
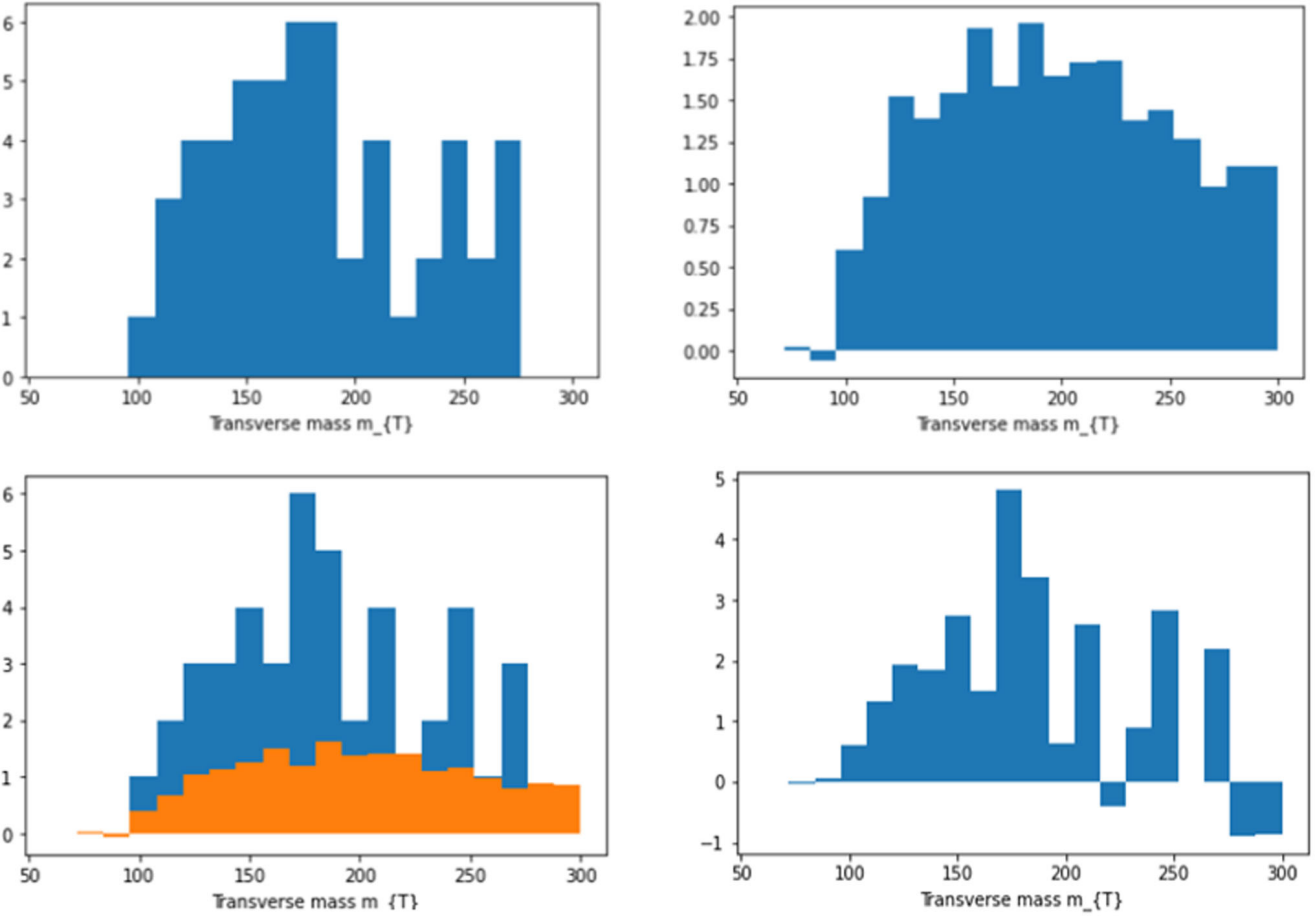
Histogram of the transverse mass of the leptons produced in data as the decay products of the 2 *W* bosons (top left), histogram of the transverse masses of lepton from the simulated background events (top right), histogram of lepton transverse mass data with simulated backgrounds (above 2 graphs combined) (bottom left), and background-subtracted histogram showing the observed Higgs signal (bottom right)

To find a signal for the Higgs boson decaying to two *W* bosons, we used a non-resonant search technique to plot a histogram of the transverse mass of the decay products of the two *W* bosons. To perform this analysis, we first selected only data events with ‘good-quality’ leptons, using similar selections to the Higgs to diphoton channel.

For this channel, we then selected pairs of leptons with different flavours and opposite charges, to reduce contributions from background processes. We also placed requirements on the transverse momenta, with the leading and subleading leptons requiring $p_{\mathrm{T}}>22$ GeV and $p_{\mathrm{T}}>15$ GeV, respectively.

Then, we applied various event-level selection criteria, including the magnitude of the missing transverse energy (MET) and the angle $\phi$ between the MET and the dilepton system, to ensure that the selected events were consistent with the $H\rightarrow WW$ signal and that events that may have arisen from background processes were rejected.

Next, we used the samples of $qq\rightarrow WW$ Monte Carlo simulations provided by the ATLAS Open Data to model the expected background contributions, scaled to match the luminosity of experimental data used. The same selections that were applied to the data were also applied to the simulation, and the transverse mass of the dilepton system in the simulated events was also plotted in a histogram. The final step was to subtract the background from the data, and this produced our Higgs signal.

Each of the steps described above is shown in Fig. 26.

#### Higgs to two *Z* bosons ($H\rightarrow ZZ$) channel

**Fig. 27:**
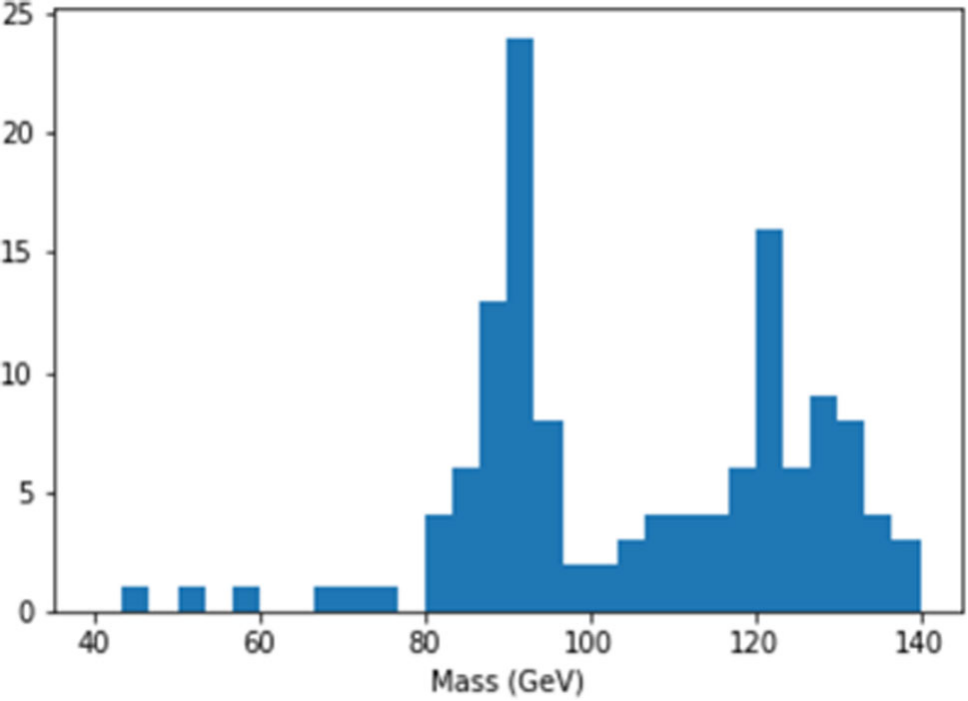
Higgs to *ZZ* channel histogram

To investigate the Higgs to *ZZ* decay channel in the four-lepton final state, we modified the code used in Sect. 6.5.2 to instead plot the invariant mass of the *Z* boson. Instead of using datasets with events in the two-lepton final state, we used data events from the datasets with events in the four-lepton final state.

Slightly different selection criteria were applied to the datasets, allowing only events with two pairs of same-flavour opposite sign leptons to target the decay of a *Z* boson. These pairs were used to reconstruct two pairs of TLorentzVectors, corresponding to the leading and trailing *Z* boson. The two reconstructed *Z* bosons were then combined to reconstruct the four-momentum of the *ZZ* system, which was used to plot the histogram of the system’s invariant mass. Figure 27 shows the results of this method using the events from Dataset D of the ATLAS Open Data four-lepton final-state data collection. There is a clear peak at 125 GeV, which is the accepted value for the invariant mass of the Higgs boson. This is evidence that the Higgs boson exists and was produced during several of the events in the dataset used.

### Discussion

After examining all our histograms, it was clear that there was a pronounced bump at 125 GeV for the $H\rightarrow \gamma \gamma$ and $H\rightarrow ZZ$ channels, the expected invariant mass we would expect from the Higgs boson, in addition to a clear Higgs signal in the $H\rightarrow WW$. Since we conducted multiple investigations through different decay channels, it solidified our evidence for observing the Higgs boson.

We ensured that we worked well as a team and divided up sections to focus our analysis on, and met regularly to share our findings and create a plan of our next steps. It also meant we could work through coding challenges together, as we were able to share similar experiences and collaborate to find a solution. We also watched some lectures and videos to enable us to understand the relevant physics before diving deep into our research, so that we could get the most out of the project.

### Acknowledgements

We would like to thank the Institute for Research in Schools, Rutherford Appleton Laboratory, and the University of Oxford for providing us with this enriching opportunity. Moreover, thank you to our teacher, Mr Carpenter, and coordinator, Dr Richard Phillips, for supervising and supporting us. In addition, a special thank you to Professor Alan Barr and his Ph.D. students for their guidance and for inspiring us to pursue this project.

## Searching for the Higgs boson through its decay into a muon-antimuon pair

By students of **King Edward VI Camp Hill School for Boys**: Rohan Desai, Yijun Chen, Amogh Shetty, Ishaan Dubey.

### Preface

*In this section, students use the ATLAS Open Data and the skills developed in all seven notebooks to design an original search for a rare Higgs boson decay:* $H\rightarrow \mu \mu$. *The students also implement several ideas in statistics, such as kernel density estimation to perform a continuous fit to a histogram, signal significance, and p-values.*

### Summary

Using Atlas Open Data [28], we searched for the predicted decay of the Standard Model (SM) Higgs boson into a muon-antimuon pair. The Large Hadron Collider (LHC) [2] provided us with data at $\sqrt{s}$ = 13 TeV from proton–proton collisions. By imposing selection criteria, we isolated events that are most likely to exhibit the characteristics of our desired decay. We reconstructed the masses of these events using the TLorentzVector class, provided in the Jupyter [7] notebooks, to calculate our invariant dimuon mass, $m_{\mu \mu }$, which we used to populate a histogram. We used Monte Carlo (MC) files from the ATLAS Open Data to simulate the backgrounds of this decay, which we then subtracted from the real data to isolate the signal. After processing 9.4 million MC events and 12.2 million data events, a peak was revealed within the range for the mass of a Higgs boson,^1^$m_{\mathrm{H}}$, at 125.66 GeV. The observed significance for a Higgs boson at $m_{\mathrm{H}}=125.38$ GeV was calculated to be 1.169$\sigma$. While not statistically significant to the level of observation, this result supports the possibility of the decay of a Higgs boson to second-generation fermions.

### Introduction

All particles are proposed to be excitations of fields—the Higgs boson is an elementary particle in the Standard Model and an excitation of the Higgs field, which gives mass to elementary particles. The Higgs field is a scalar field; therefore, its associated boson is scalar and has a spin of zero. The addition of this field allows spontaneous symmetry breaking of the electroweak interaction, giving mass to many particles via the Higgs Mechanism.

The Higgs field can be analogised to crossing an infinite, flat field of snow. The following scenarios are possible:

- Skiing across the top—this is analogous to a high-energy particle not interacting with the Higgs field. It does not sink into the ‘snow’ as it is travelling at the speed of light and therefore has no mass.
- Walking in snowshoes—they will sink into the ‘snow’ as they travel slower than before. This is like a particle with some mass as this person somewhat interacts with the field.
- Walking regularly—this person will sink deeply into the field, as they are travelling very slowly and with little energy. This represents a particle with greater mass that interacts strongly with the field.

Just as the snowfield is made up of tiny, individual snowflakes, the Higgs field gives rise to many Higgs boson excitations, which give mass to elementary particles.

The Higgs boson was discovered in 2012 by the ATLAS [1] and CMS [29] experiments at the Large Hadron Collider (LHC) [2] at 5.9$\sigma$ significance [41]. It is measured to have an invariant mass of $125.38\pm 0.14\hbox { GeV}$ [39], and is found to be consistent with the predicted properties for the Higgs boson by Peter Higgs et al. in 1964 [42]: Even (positive) parity, no electric charge, no colour charge, zero spin, and zero strong force interaction. Even the Higgs branching ratios have agreed with those predicted. The first evidence of fermion interactions with the Higgs field was through Higgs decay to tau particles, which was observed in the combination of ATLAS and CMS results performed at the end of Run 1 at the LHC, and later remeasured at a higher significance [43].

The Higgs boson is very unstable, with a lifetime of $1.6\times 10^{-22}$ seconds [44], which means it decays almost immediately, making it difficult to find. By measuring decay rates to different particles, the predicted mechanism by which they acquire mass can be tested. Measurements performed so far have focused on Higgs boson interactions with the most massive particles, such as the *W* and *Z* bosons, and only with particles from the most massive generation, the top and bottom quarks and the tau lepton. The interaction of the Higgs boson with lighter particles, such as muons, has so far not been observed. Measuring the full spread of Higgs boson interactions is critical to test if the Higgs mechanism can explain the full range of particle masses.

**Fig. 28:**
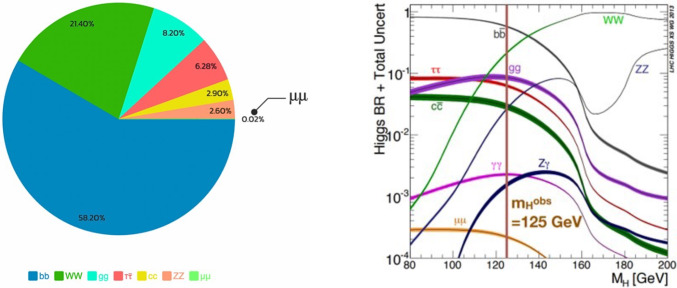
The branching ratios of a 125.38 GeV Higgs (left), created using data from [46], the branching ratios of the Higgs with respect to the mass of the Higgs (right), from [47]

The Standard Model predicts several rare Higgs boson decay channels which have not yet been observed. Among these are decays to second-generation leptons and quarks, e.g. $H\rightarrow \mu \mu$, and $H\rightarrow Z\gamma$. The focus of this project is on one of the rarest decays: the Higgs boson into a dimuon pair ($H\rightarrow \mu \mu$). The expected branching fraction for the decay of the Higgs boson into a pair of muons at $m_{\mathrm{H}}=125.38$ GeV is $B(H\rightarrow \mu \mu ) = 2.18 \times 10^{-4}$ [45]. Figure 28 displays this statistic more visually. Other more prevalent decays have significantly higher branching fractions, making them easier to detect.

Only one in five thousand Higgs bosons is predicted to decay to muons. And, like a needle in a mountain of needles, for every predicted decay of a Higgs boson to muons at the LHC, there are a thousand pairs of muons that mimic our desired signal [45]. This background from other particles makes isolating the Higgs boson decay to muons extremely difficult. Therefore, the efficacy of event selection and simulation is paramount. The $H\rightarrow \mu \mu$ decay offers the best opportunity to measure the Higgs interaction with second-generation fermions at the LHC, providing new insights into the origin of mass for different generations.

### Methods

The ATLAS experiment [1] is located at the LHC [2], which collides protons at high speeds and uses a set of complex detectors to measure the outcome. Data from these collisions are organised into ’events’, some of which have been made available to the public. We used files from the ’ATLAS Open Data’ [28] to conduct our investigation. The ‘bump hunt’ method that we employed is commonly used for measurements at CERN - using the law of conservation of energy, the Higgs boson can be found by reconstructing the invariant masses of its decay products ($m_{\mu \mu }$). After selecting appropriate events using the selection criteria in Table 1, each dimuon invariant mass is added to a histogram. This process was repeated millions of times, so the final histogram should have a higher frequency around the mass of a Higgs boson (125.38 GeV)—a ‘bump’ suggesting existence of the $H\rightarrow \mu \mu$ decay. The same process can then be carried out using simulated data for the backgrounds to this decay, and this background is subtracted from the data to enhance the visibility of our bump. A statistical analysis is then performed to quantify this evidence.

**Table 1:** Detailed explanation of all selection criteria used to filter the events

Selection criteria	Explanation
Exactly two muons with opposite charge	These criteria are used to select events that contain a muon-antimuon pair
$p_{\mathrm{T}}^{\mu , \textrm{leading}}> 27$ GeV	Used to select high-energy muons, which are more likely to be produced in decays of heavy particles like the Higgs boson
$p_{\mathrm{T}}^{\mu , \textrm{subleading}}> 15$ GeV	This ensures that both muons in the event are detected with a reasonable efficiency, as they must have a high energy
$-2.7 \le \eta ^{\mu } \le 2.7$	Used to select muons in the central region of the detector. Muons produced at larger angles from the beamline are more likely to be from background noise and are also detected with less accuracy
No *b*-tagged jets	*b*-tagging is a technique used to identify jets which contain a *b* quark, which are a large background to $H\rightarrow \mu \mu$. These are removed to increase the signal-to-noise ratio
$p_{\mathrm{T}}^{\mathrm{jets}} > 25$ GeV	Used to select jets that are produced with high energy while minimising the contribution from low-energy background events, because the $H\rightarrow \mu \mu$ decay must produce high-energy particles
$-2.5 \le \eta ^{\mathrm{jets}} \le 2.5$	Used to identify the jets produced in the central region of the detector. Jets that fall into other regions are less accurately recognised and are more likely to be background noise

#### Event selection

To find this extremely rare decay, we used selection criteria: a set of filters applied to the data to distinguish the relevant signal from background data. After extensive research [48, 49], we produced the selection criteria shown in Table 1 to identify and isolate the specific events where this dimuon decay has occurred. The same selection criteria were used to filter the real and simulated events.

#### Simulated events

We simulated the collisions of particles in the LHC using files from the 13 TeV ATLAS Open Data set [28]. Monte Carlo (MC) simulation files are used to simulate the behaviour of subatomic particles produced by the LHC. We used electroweak diboson and Higgs MC files from the Open Data, which we believed would mimic the expected background of the $H\rightarrow \mu \mu$ decay. The simulated events were filtered through the same selection criteria as in Table 1.

#### Invariant mass reconstruction and plotting

**Fig. 29:**
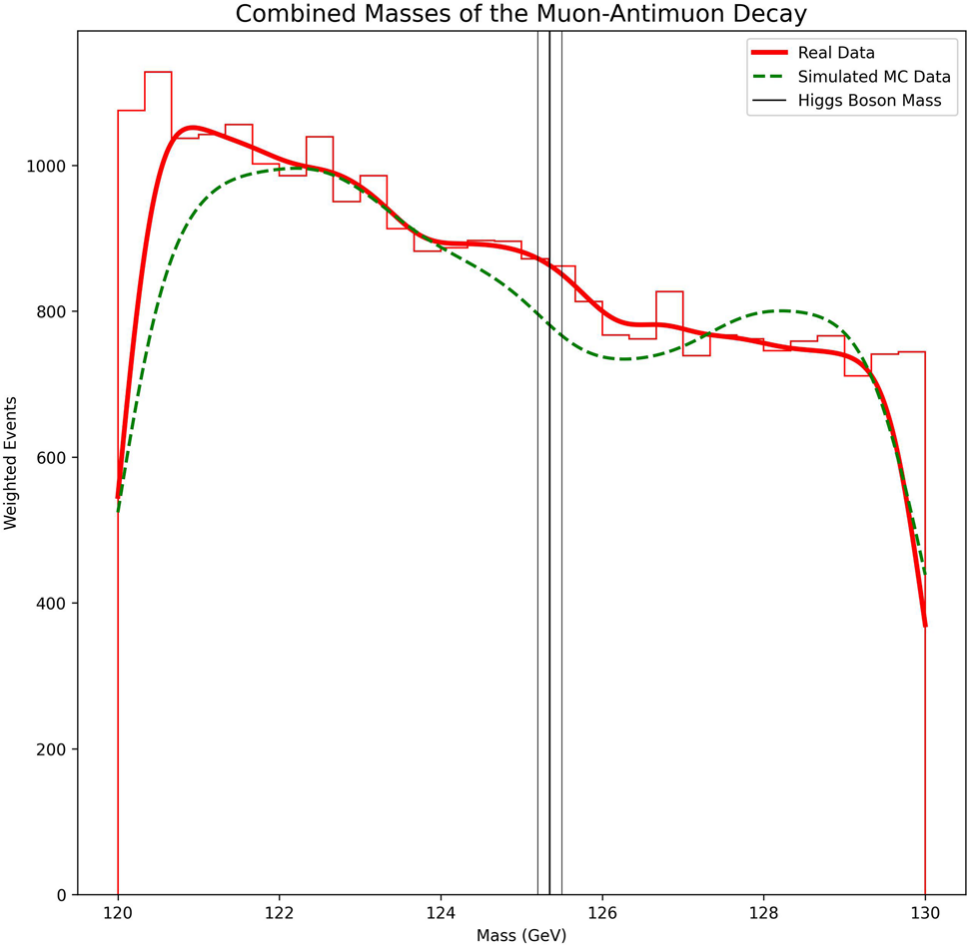
The line formed after using a Gaussian KDE to transform the histogram of the real dimuon masses, in red. The green line shows the same for Monte Carlo simulated events. The *y*-axis displays frequency, with the simulated graph being scaled up to have the same proportions as the real data

Using the selection criteria in Table 1, we found the data events most likely to exhibit the characteristics of the $H\rightarrow \mu \mu$ decay and background MC events that mimic $H\rightarrow \mu \mu$. Using the implementation of ROOT’s [6] TLorentzVector class in the training notebooks, we used the transverse momentum, pseudorapidity, azimuthal angle and energy of these events to reconstruct the four-momentum of our desired decay. The notebook implementation of the ROOT SetPtEtaPhiE and M functions allowed us to easily calculate $m_{\mu \mu }$ for each event and add it to a histogram, forming the red histogram of ’real data’ in Fig. 29. We used ‘kernel density estimation’ (KDE) to transform our discrete histogram into a continuous line, improving our ability to find patterns in the distribution. The Gaussian distribution was our “kernel”, and we used Scott’s rule to calculate the bandwidth of the kernel, giving us an appropriate resolution for the data.

The same process was repeated for the simulated events; this formed the red and green lines for real and simulated data, respectively, in Fig. 29. As there was a difference in the number of real and simulated events produced, we scaled the simulated graph up to have the same normalisation as the distribution of real events. This step helped us compare the simulated background data to real data, allowing us to identify events consistent with the expected behaviour of $H\rightarrow \mu \mu$ decay and remove events that are the result of other background processes. Any difference in the two lines in Fig. 29 suggests a deviation from the expected behaviour, providing evidence for the $H\rightarrow \mu \mu$ decay.

### Results

**Fig. 30:**
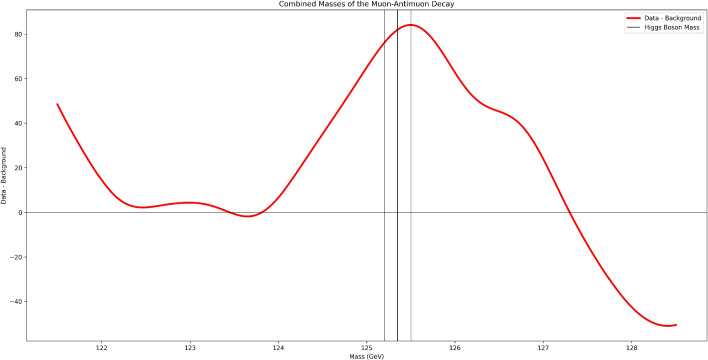
Graph of weighted data events with simulated backgrounds subtracted

After applying the event selections in Table 1 to the reconstructed data and simulation, as shown in Fig. 29, we were able to create Fig. 30 by subtracting the simulated background from the data, therefore isolating the signal. In total, we processed 12.2 million real data events and 9.4 million MC events, with the latter scaled up to have equal luminosity as the data. Our selection criteria were able to filter these background events to approximately 35% of their original number. There is a significant spike at 125.5 GeV, which lies in the 1$\sigma$ confidence interval for the Higgs mass (125.38±0.15 GeV). Due to the rarity of this decay, we were unable to isolate all the signal from the background, leaving us with bumps/ troughs at m $\simeq$ 121 GeV and 128 GeV, respectively. The bump in MC data in Fig. 29 between 128 and 129 GeV appears to show the presence of another background decay that survived our selection criteria. This highlights the challenges in isolating rare events from background noise. Ideally, we would investigate this decay in order to possibly isolate the signal data further, which would produce a clearer spike.

**Fig. 31:**
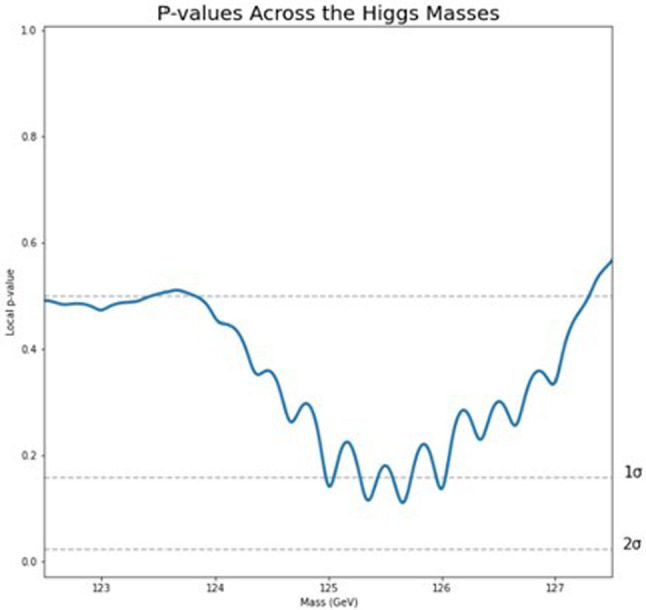
Plot of observed local p-values of results at a range of test masses for the Higgs boson. Dotted lines show the corresponding 1$\sigma$ and 2$\sigma$ values

From our results, we conducted a statistical analysis to find the *p* values (*p*) and the sigma values ($\sigma$), testing the Higgs boson mass hypothesis over an interval. We used the Gaussian function to calculate the error in our methods to calculate the data and background for each mass, then we used the data and background values from Fig. 29 to calculate the $\sigma$ and *p* value. This process was repeated between 122 and 128 GeV, creating Fig. 31 which displays a *p* value against the tested Higgs mass. At $m_{\mathrm{H}}=125.38$ GeV, the accepted value for the Higgs mass, our observed $\sigma =1.169$ ($p=0.121$). However, we found at $m_{\mathrm{H}}=$125.66 GeV, $\sigma = 1.224$ ($p=0.111$). The periodic fluctuation in our graph was a point of interest, something we hope to understand and improve on in the future. Further data could be processed to give a larger sigma value, as well as exploring the questions outlined in Sect. 7.5.

### Discussion

From the results of our research, we have found evidence in support of the existence of a Higgs decay into a muon-antimuon pair. Figures 29 and 30 show that there was an excess of events around 125 GeV, which is consistent with the expected signal from the Higgs boson decay. More precise results would be needed to confirm this as a discovery. The results from different Higgs decay modes could be combined to improve the precision of the measurements and provide a more complete understanding of the Higgs boson’s properties.

Another improvement to this project would be to divide the decay into its production categories, a method that previous searches have used [48, 50]. Specifically, there are four exclusive categories of Higgs boson production: Gluon-gluon fusion (ggf), association with vector boson (VH), vector boson fusion (VBF), and association with the top quark and antiquark pair (ttH). VBF has shown to be dominant out of the four; its events could be investigated exclusively to obtain better results. Our next steps would be to apply specific selection criteria for each of these categories to refine our search for the dimuon pair, likely resulting in a bigger peak. However, it would be difficult to separate events to that degree with our dataset, as that level of refining would leave each category with a low number of events, resulting in larger uncertainty and less reliable results.

Although the ATLAS Open Data were very useful in providing us with the necessary information and resources to develop our skills, we found that some of the data we would have liked were not provided. For our decay ($H\rightarrow \mu \mu$), we would have liked to have been provided with a simulation of the $H\rightarrow \mu \mu$ signal and certain specific backgrounds, but these were not available. Instead, we had to compromise the accuracy of our results by combining similar processes. An alternative would be to simulate this background ourselves, using open-source software such as MadGraph or Delphes. However, MadGraph does not model particle interactions with the ATLAS detector, and while Delphes does, it is not approved by ATLAS. Therefore, there was no way to accurately simulate the background ourselves, highlights a limitation of the ATLAS Open Data to motivated student researchers. Although some alternatives would have given better simulation results than ours, none would be comparable to those used at CERN. Furthermore, using machine learning algorithms such as XGBoost to identify *b*-tagged jets could more accurately isolate the signal data.

When evaluating our project, we found that we had initially overlooked the process of converting the histogram into a continuous curve. We used a simple kernel density estimation and found a width using Scott’s rule, but after further research we understood that we should have controlled this process further. One ATLAS paper stated “The width of the Gaussian component of the double-sided Crystal Ball function varies between 2.6 and 3.2 GeV depending on the category” [50]. A width between 2.6 and 3.2GeV would have given a less sensitive but more appropriate resolution for this investigation. Our function also took into account the edges of the histogram, as seen in 29, where the graphs fall significantly on each side. If we were to repeat this process, a more appropriate function would be used.

With our observed $\sigma =1.224$, this project supports the prediction of the $H\rightarrow \mu \mu$ decay and provides evidence for the decay of the Higgs boson to second-generation fermions. Although the ATLAS Open Data was extensive, an extension to the data provided would have resulted in a better result.

### Acknowledgements

We would like to thank the Institute of Research in Schools, Rutherford Appleton Laboratory, and Oxford University for providing the data and learning modules to develop our ideas and conduct research, as well as the opportunity to present our findings at the IRIS Student Research Conference in London. We would also like to thank William Murray from Rutherford Appleton Laboratory for helping us to conduct a statistical analysis to find our sigma values. Finally, we would like to thank our school teacher Daniel Redshaw for supervising the project.

## Applications of the XGBoost machine learning algorithm in particle physics

By students of **King Edward VI Camp Hill School for Boys**: Shizhe Liu, Sasan Hapuarachchi, Pruthvi Shrikaanth, William Shi, Joel Regi.

### Preface

*In this section, students utilise the ATLAS Open Data and skills developed in all seven notebooks to explore the potential of machine learning in particle physics analyses. Students compare traditional ‘cut and count’ methods to the output of the XGBoost classifier for different Standard Model and Beyond Standard Model processes. Students evaluate the performance of the machine learning algorithm using ROC curves and the Approximate Mean Significance metric.*

### Abstract

The rise in technological developments in artificial intelligence has opened up new avenues of exploration at the intersection of machine learning (ML) and particle physics. We evaluated the potential of the XGBoost (ML) algorithm, a powerful gradient-boosted decision tree classification algorithm, to streamline the process of identifying rare particle decays. We achieved this by comparing the performance of XGBoost in four different classification problems in particle physics with the performance of existing classification methods, such as the application of strict cuts. We found that while in some cases the algorithm provided near-perfect prediction results, the algorithm was overly rigorous in other cases, leading to large numbers of signal events being dismissed as background events by the algorithm.

### Introduction

#### Motivation

Currently, the process of searching for rare particle decays presents a significant challenge for particle physicists, as these decays can only be found in a tiny proportion of the millions of events picked up by the sensors in particle detectors. The emergence of the XGBoost, a powerful classification algorithm, has the potential to aid particle physicists in resolving this challenge, as it may provide a better alternative to existing methods in identifying the events that contain elusive particle decays.

However, there are limited studies on the performances of the XGBoost algorithm in particle physics classifications. Therefore, we aim to address this gap by identifying the specific areas within particle physics where XGBoost excels and to compare its performance with existing methods, such as applying stringent cuts. We evaluated the algorithm’s classification performance in four different event types:

1. Higgs boson events;
2. Supersymmetry events;
3. Beyond standard model $Z^{'}$ events;
4. Kaluza–Klein graviton events.

#### XGBoost classification algorithm

As advances in artificial intelligence continue to be made, increasingly powerful machine learning algorithms are being developed at a rapid pace, and the XGBoost algorithm is an example of a classification algorithm that has arisen as a result of this technological revolution. It uses gradient boosted decision trees to provide accurate classification results. ‘Boosting’ is a technique where new models correct errors made by previous ones, and are added one by one until no further improvements occur. ‘Gradient boosting’ allows models to predict the errors made by the previous models, to help provide an accurate result. The main advantages of XGBoost are its excellent speed and accuracy, due to its ability to discern subtle patterns in the data, in providing accurate predictions that may prove invaluable when performing particle physics classifications [51].

#### AMS metric

We evaluated the performance of the XGBoost classifier algorithm in carrying out the different categories of classification using the Approximate Median Significance (AMS) metric. When providing the true positive and false positive rates of the classification, the AMS metric uses the Wilks Theorem to compare the probabilities of observing the signal+background hypothesis and the background only hypothesis, to return an overall figure which represents the performance of the classifier. Therefore, AMS provided us with a standardised way of assessing the XGBoost algorithm in the different applications tested [52].

### Results

#### Higgs boson event classification

**Fig. 32:**
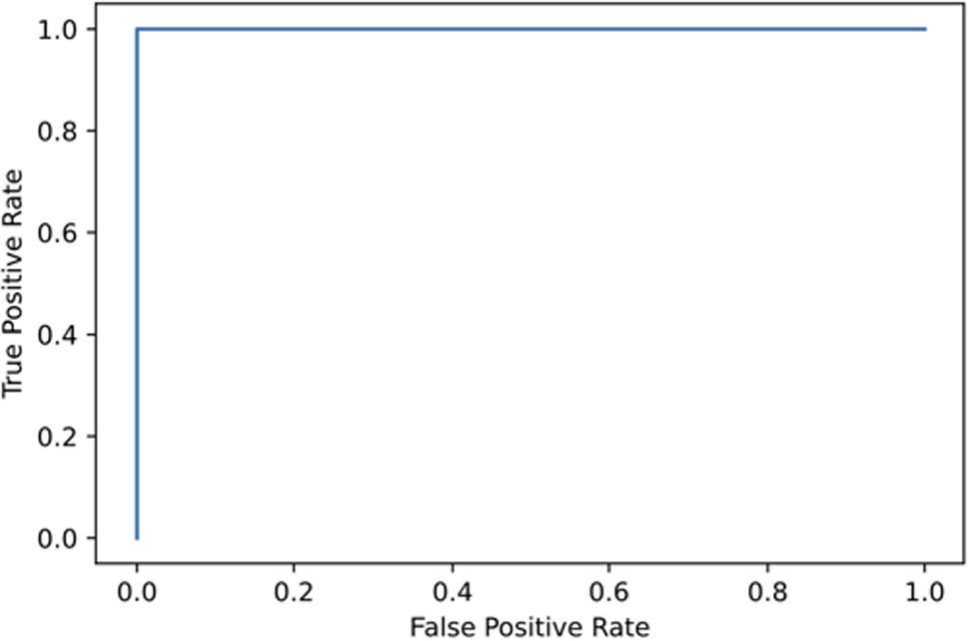
Higgs boson classification ROC curve

Prior advancements in particle physics have revealed that it is possible to represent every particle as a wave in quantum fields. One such field is the Higgs field, which suggests that there would be a particle associated with this field, the Higgs boson [53]. We wrote a programme that trained the XGBoost algorithm to classify Higgs boson events using ATLAS Open Data samples containing over 400,000 events, including a mixture of simulated signal and background events [54], and tested it with another set of 400,000 events. All the test events that the XGBoost classifier had classified as signal events were, indeed, a real signal event. This yields an AMS score of infinity, which suggests that the XGBoost algorithm performed very well in classifying Higgs boson events. This can be reinforced by observing the ROC curve, which compares the true positive rate (TPR) against the false positive rate. As evident in Fig. 32, the TPR initially increases rapidly, further suggesting that the XGBoost is accurate.

#### Beyond standard model $Z^{'}$ event classification

The second type of particle we looked at is the $Z^{'}$ boson hypothesised by the Topcolor model—a model for electroweak symmetry breaking, in which a top anti-top pair forms a composite Higgs boson. In this model, a $Z^{'}$ boson is predicted to exist, which we decided to investigate via the XGBoost classifier.

**Fig. 33:**
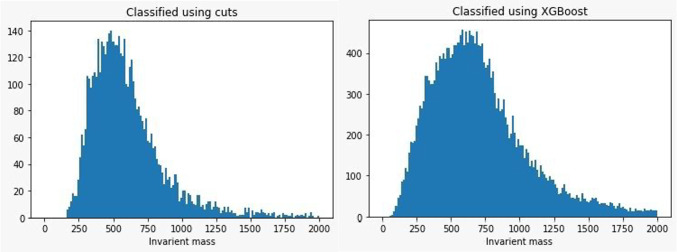
The invariant mass of $Z^{'}$ events classified using strict cuts (left), and $Z^{'}$ events classified using the XGBoost algorithm (right)

To find these particles, we looked at the decay channel; in this case, the $Z^{'}$ decays into a top-antitop pair in events with a single charged lepton, large-radius (large-R) jets and missing transverse momentum [17]. The lepton must have a transverse momentum > 30 GeV, missing transverse energy > 20 GeV, a small radius (small-R) jet close to the lepton, a large-R jet passing the top tagging requirements (mass > 100 GeV, N-subjettiness ratio < 0.75), etc.

Finally, we plotted the histograms of the $Z^{'}$ invariant masses from the cut-based analysis (Fig. 33 (left)) and the results of the XGBoost classifier (Fig. 33 (right)).

#### Kaluza–Klein graviton event classification

The third type of particle we looked at is a Kaluza–Klein graviton hypothesised in the Randall–Sundrum model [55], a model for gravity in which gravity propagates through warped extra dimensions. Similarly to atoms having excited states or low energy states, particles can have corresponding Kaluza-Klein states where the particle has extra mass in other dimensions [56].

To find the Kaluza–Klein graviton, we searched for the particles into which it decays, in this case a $\gamma \gamma$ pair. To find this particle, we performed a bump hunt in photons with transverse energies over 20 GeV [55]. To do this, we made the following cuts to the ATLAS Open Data set [26]: the event must have two photons, it must activate the photon trigger, and both photons must have a transverse energy greater than 20 GeV. Once we obtained our data points, we subtracted any that could have been produced by a $H\rightarrow \gamma \gamma$ decay and plotted a graph of invariant mass against frequency using a fitting function.

**Fig. 34:**
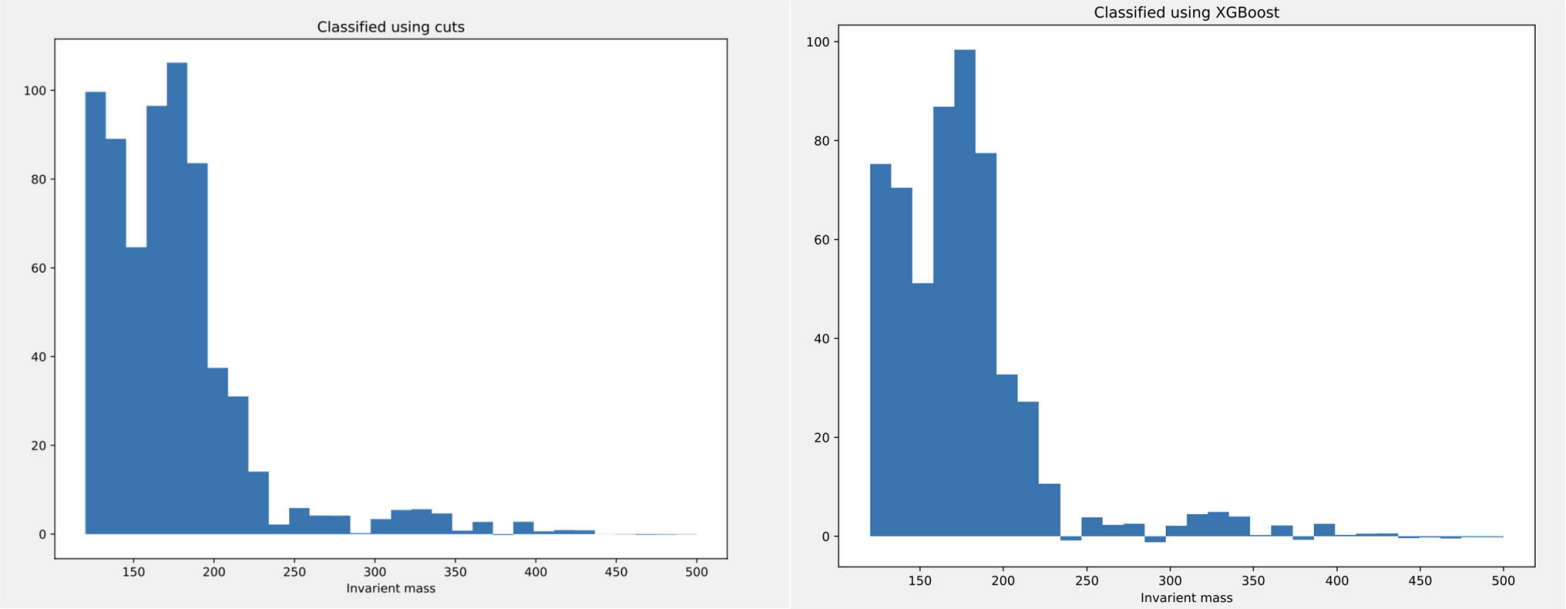
Kaluza–Klein graviton events classified using restrictive cuts (left), and Kaluza–Klein graviton events classified using the XGBoost algorithm (right)

XGBoost classifiers were trained on ATLAS Open Data samples containing information (e.g. number of leptons, transverse mass, jet) to identify events which had good-quality photons and isolated photons, respectively—this problem required two classifiers. The models were then used in parallel to identify events which contained the decay of the Kaluza–Klein graviton, which typically had both good photons and photon isolation. The events classified by XGBoost were then plotted on a histogram shown in Fig. 34 (right) and compared with the original cuts-based histogram shown in Fig. 34 (left) to see the performance of the classification. The AMS values were 866.8 for the good photon classification and 866.7 for the photon isolation classification.

#### Supersymmetric event classification

‘Supersymmetry’ (SUSY) is the hypothesis that every fermion has a partner boson with different spin properties, where fermions have half-integer spin values and bosons have integer spin values [57]. We can search for supersymmetric particles by examining pairs of particles created from collisions in the LHC. To do this, we used Python to examine the ATLAS Open Data [28] and make ’cuts’ on it. These cuts filter out data that we do not need, leaving us with a subset of data that is much more useful for examining supersymmetric events.

**Fig. 35:**
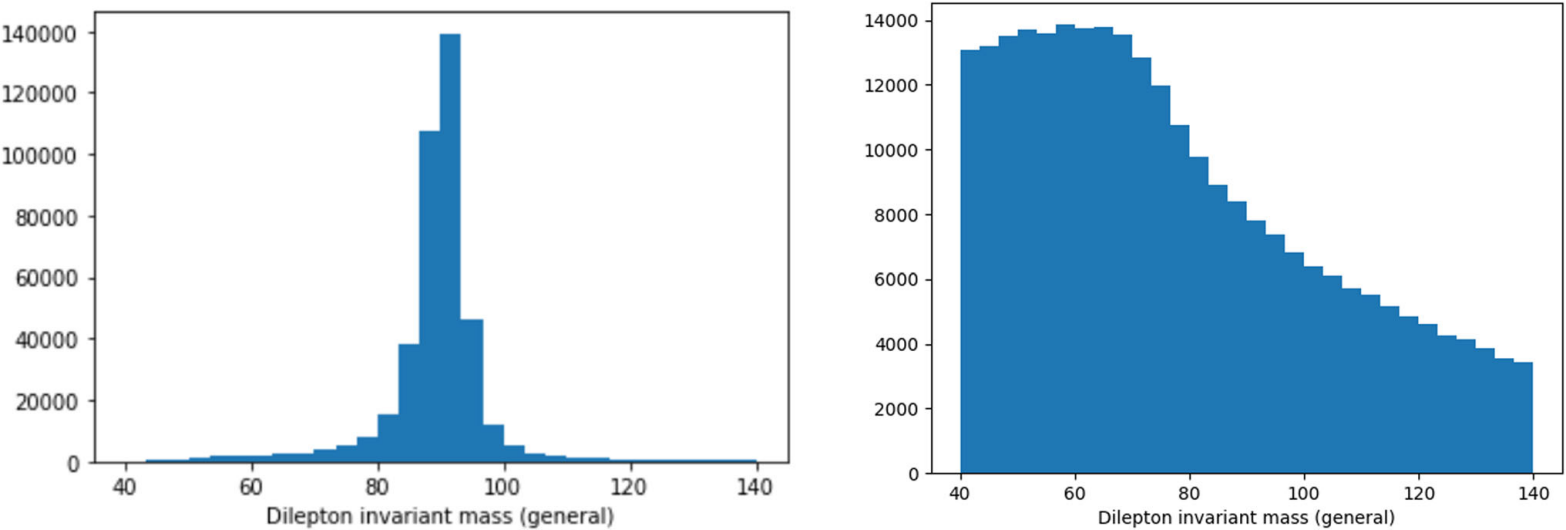
SUSY events general classification using restrictive cuts (left), and SUSY events general classification using the XGBoost algorithm (right)

To carry out this investigation, we first used uproot to load the ATLAS Open Data and initialised our histograms to be plotted later. After this, we extracted all the information we needed to make our cuts from the data and stored it. Then, we set up variables to store the four-momentum of particles by creating four-vectors of their kinematics. A series of cuts were made, starting with selecting only collisions between electrons and muons in pairs of the same type and opposite charge. We then selected events where each particle had a minimum momentum, which we decided by following recommendations from the ATLAS experiment at CERN. Following this, we calculated the momentum of leading and trailing leptons, and manipulated their four-vectors to find the dilepton invariant mass. We then made further cuts based on the invariant mass and the accuracy of detected jets. Next, we sorted these leptons into categories based on the magnitude of their invariant mass and the event’s MT2 variable [58], which is related to the transverse mass of unseen particles. Finally, we plotted the distributions of the dilepton invariant mass with general (least strict), loose and tight requirements on dilepton invariant mass and MT2 values.

**Fig. 36:**
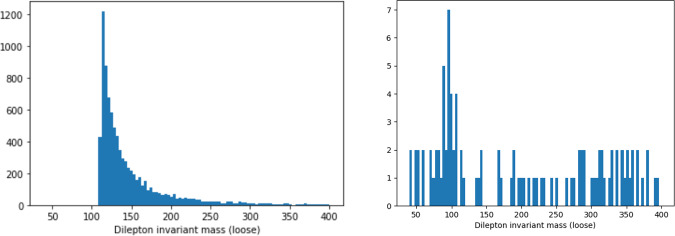
SUSY events loose classification using restrictive cuts (left), and SUSY events loose classification using the XGBoost algorithm (right)

**Fig. 37:**
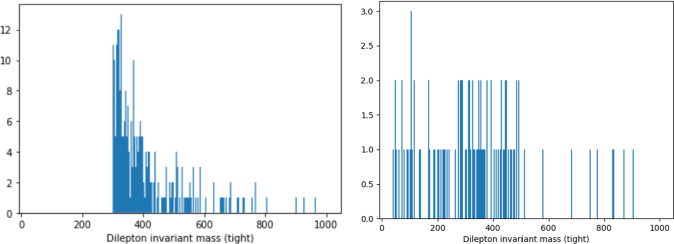
SUSY events tight classification using restrictive cuts (left), and SUSY events tight classification using the XGBoost algorithm (right)

After obtaining these results, we stored them in a.csv file. We then used the XGBoost machine learning algorithm, training it on half of our data, and testing it on the other half to see how well it matched the results of the cut-based analysis. To find the precision of our ML algorithm, rather than simply calculate the proportion of predictions that were ’correct’ or within an acceptable range, we used the AMS metric, defining a function that implements the AMS metric and then calculates it for each category (general, loose, tight). The graphs plotted by the XGBoost algorithm are given in Figs. 35, 36 and 37. The algorithm produced graphs, and AMS values $\sim$1.689 (loose) and $\sim$1.192 (tight), while the general category had an AMS value of $\sim$603.226. This may have been a result of using a smaller dataset as this would have resulted in a weaker model. The comparison of the graphs shows us that our loose events classification predicted by the XGBoost algorithm shown in Fig. 36 (right) is most similar to the loose events classification made using cuts shown in Fig. 36 (left), sharing a shape with the tight classification graphs shown in Fig. 37. Hence, we found that loose cut requirements were the best for building an accurate model to detect supersymmetric particles, although they may lead to more false positives than desirable when compared to tight requirements.

### Discussion

From BSM particles to delving into supersymmetry, we have explored the performance of the XGBoost machine learning algorithm across a wide range of cutting-edge classification problems in particle physics.

Our results revealed significant variations in the performance of the XGBoost algorithm across our tested range of particle physics classification problems. In some areas, such as the Higgs boson classification, the XGBoost gave perfect or near-perfect prediction results. However, in other cases, it was clear that the XGBoost displayed excessive rigidity, leading substantial portions of the signal data to be excluded and dismissed as background data, resulting in lacklustre distributions, as seen in our exploration of SUSY.

The XGBoost is a supervised learning algorithm, and so relies on labelled datasets. Therefore, the algorithm works best in classifications where the selection criteria are well defined, as it allows accurately labelled training datasets to be generated. In these cases, researchers may not feel the full benefit of the XGBoost, as the algorithm will only ever (at best) replicate a prior cut-based analysis. However, this is an issue that we hope to address in our future work by exploring the potential of deep learning models to identify the optimal selection criteria for particle decay classifications which have very few selection criteria identified so far.

## Conclusion

In this article, a variety of original research projects performed by UK secondary school students using the ATLAS Open Data and the repository of training resources developed by the authors have been presented. Such student research output shows that secondary school students are capable of meaningfully engaging with public releases of LHC data, presupposing no prior knowledge or experience. With sufficient time, training and support, it has been demonstrated that it is possible for high school students to interact with the data presented in the same format and using the same analysis techniques as physics researchers. Additionally, it has been shown that, with correctly structured training, students can produce entirely original works of research, and often independently arrive at questions and ideas that exist at the cutting-edge of particle physics research. Key to such successes is the structure of the training materials; they must presuppose no prior knowledge and present new information in a step-wise manner (preferably in a variety of formats), coding examples should be thoroughly commented with interleaved exercises to consolidate learning, hints and solutions should be available throughout to prevent frustration due to students becoming ’stuck’. Crucially, off-ramps from the training should be provided at each level, so individual teachers can tailor the project to the particular group of students, and the time and resources available. In conclusion, the student research presented in this article makes a strong case for the value of the public release of LHC data, and for the ongoing support for the ATLAS Open Data project.

## Data Availability

The data and simulation that support the findings of this study are openly available in the ATLAS Open Data repository, at http://doi.org/10.7483/OPENDATA.ATLAS.GQ1W.I9VI, http://doi.org/10.7483/OPENDATA.ATLAS.B5BJ.3SGS, http://doi.org/10.7483/OPENDATA.ATLAS.2Y1T.TLGL and http://doi.org/10.7483/OPENDATA.ATLAS.FRWJ.4ZQU. The manuscript has associated data in a data repository.

## Footnotes

1 At the time of writing, it is accepted that $m_{\mathrm{H}}$ = 125.38±0.14 GeV [39]. Recent research has shown that $m_{\mathrm{H}}$ = 125.11±0.11 GeV may be possible [40].
